# Studies of the effectiveness of transport sector interventions in low‐ and middle‐income countries: An evidence and gap map

**DOI:** 10.1002/cl2.1203

**Published:** 2021-11-27

**Authors:** Suchi Kapoor Malhotra, Howard White, Nina Ashley O. Dela Cruz, Ashrita Saran, John Eyers, Denny John, Ella Beveridge, Nina Blöndal

**Affiliations:** ^1^ Campbell South Asia New Delhi India; ^2^ Campbell Collaboration New Delhi India; ^3^ Consultant, Campbell Collaboration Las Pinas Philippines; ^4^ Independent Consultant Somerset UK; ^5^ Campbell Collaboration Ottawa Canada; ^6^ Concord Consulting Copenhagen Denmark

## Abstract

**Background:**

There are great disparities in the quantity and quality of infrastructure. European countries such as Denmark, Germany, Switzerland, and the UK have close to 200 km of road per 100 km^2^, and the Netherlands over 300 km per 100 km^2^. By contrast, Kenya and Indonesia have <30, Laos and Morocco <20, Tanzania and Bolivia <10, and Mauritania only 1 km per 100 km^2^. As these figures show, there is a significant backlog of transport infrastructure investment in both rural and urban areas, especially in sub‐Saharan Africa. This situation is often exacerbated by weak governance and an inadequate regulatory framework with poor enforcement which lead to high costs and defective construction.

The wellbeing of many poor people is constrained by lack of transport, which is called “transport poverty”. Lucas et al. suggest that up to 90% of the world's population are transport poor when defined as meeting at least one of the following criteria: (1) lack of available suitable transport, (2) lack of transport to necessary destinations, (3) cost of necessary transport puts household below the income poverty line, (4) excessive travel time, or (5) unsafe or unhealthy travel conditions.

**Objectives:**

The aim of this evidence and gap map (EGM) is to identify, map, and describe existing evidence from studies reporting the quantitative effects of transport sector interventions related to all means of transport (roads, rail, trams and monorail, ports, shipping, and inland waterways, and air transport).

**Methods:**

The intervention framework of this EGM reframes Berg et al's three categories (infrastructure, prices, and regulations) broadly as infrastructure, incentives, and institutions as subcategories for each intervention category which are each mode of transport (road, rail trams and monorail, ports, shipping, and inlands waterways, and air transport). This EGM identifies the area where intervention studies have been conducted as well as the current gaps in the evidence base.

This EGM includes ongoing and completed impact evaluations and systematic reviews (SRs) of the effectiveness of transport sector interventions. This is a map of effectiveness studies (impact evaluations). The impact evaluations include experimental designs, nonexperimental designs, and regression designs. We have not included the before versus after studies and qualitative studies in this map. The search strategies included both academic and grey literature search on organisational websites, bibliographic searches and hand search of journals.

An EGM is a table or matrix which provides a visual presentation of the evidence in a particular sector or a subsector. The map is presented as a matrix in which rows are intervention categories (e.g., roads) and subcategories (e.g., infrastructure) and the column outcome domains (e.g., environment) and subcategories as (e.g., air quality). Each cell contains studies of the corresponding intervention for the relevant outcome, with links to the available studies. Included studies were coded according to the intervention and outcomes assessed and additional filters as region, population, and study design. Critical appraisal of included SR was done using A Measurement Tool to Assess Systematic Reviews (AMSTAR ‐2) rating scale.

**Selection Criteria:**

The search included both academic and grey literature available online. We included impact evaluations and SRs that assessed the effectiveness of transport sector interventions in low‐ and middle‐income countries.

**Results:**

This EGM on the transport sector includes 466 studies from low‐ and middle‐income countries, of which 34 are SRs and 432 impact evaluations. There are many studies of the effects of roads intervention in all three subcategories—infrastructure, incentives, and institutions, with the most studies in the infrastructure subcategories. There are no or fewer studies on the interventions category ports, shipping, and waterways and for civil aviation (Air Transport).

In the outcomes, the evidence is most concentrated on transport infrastructure, services, and use, with the greatest concentration of evidence on transport time and cost (193 studies) and transport modality (160 studies). There is also a concentration of evidence on economic development and health and education outcomes. There are 139 studies on economic development, 90 studies on household income and poverty, and 101 studies on health outcomes.

The major gaps in evidence are from all sectors except roads in the intervention. And there is a lack of evidence on outcome categories such as cultural heritage and cultural diversity and very little evidence on displacement (three studies), noise pollution (four studies), and transport equity (2). There is a moderate amount of evidence on infrastructure quantity (32 studies), location, land use and prices (49 studies), market access (29 studies), access to education facilities (23 studies), air quality (50 studies), and cost analysis including ex post CBA (21 studies).

The evidence is mostly from East Asia and the Pacific Region (223 studies (40%), then the evidence is from the sub‐Saharan Africa (108 studies), South Asia (96 studies), Latin America & Caribbean (79 studies). The least evidence is from Middle East & North Africa (30 studies) and Europe & Central Asia (20 studies). The most used study design is other regression design in all regions, with largest number from East Asia and Pacific (274). There is total 33 completed SRs identified and one ongoing, around 85% of the SR are rated low confidence, and 12% rated as medium confidence. Only one review was rated as high confidence. This EGM contains the available evidence in English.

**Conclusion:**

This map shows the available evidence and gaps on the effectiveness of transport sector intervention in low‐ and middle‐income countries. The evidence is highly concentrated on the outcome of transport infrastructure (especially roads), service, and use (351 studies). It is also concentrated in a specific region—East Asia and Pacific (223 studies)—and more urban populations (261 studies). Sectors with great development potential, such as waterways, are under‐examined reflecting also under‐investment.

The available evidence can guide the policymakers, and government‐related to transport sector intervention and its effects on many outcomes across sectors. There is a need to conduct experimental studies and quality SRs in this area. Environment, gender equity, culture, and education in low‐ and middle‐income countries are under‐researched areas in the transport sector.

**Abbreviations**
AMSTAR: Assessing the Methodological Quality of Systematic Reviews
DID: difference‐in‐difference
EGM: evidence and gap map
ICORSI: Independent Council for Road Safety International
IIT‐Delhi: Indian Institute of Technology
ITPD: Institute of Transportation and Development Policy
IV: instrument variable
LMICs: low‐ and middle‐income countries
PICOS: Population, Intervention, Comparison, Outcomes and Study design
PSM: propensity score matching
RCT: randomised controlled trial
RDD: regression discontinuity design
SDG: sustainable development goal

**Plain language summary**

**The evidence base for the impact of transport is unevenly distributed and under‐reviewed**

This evidence and gap map (EGM) shows the available evidence, and gaps in the evidence, on the effectiveness of transport sector intervention in low‐ and middle‐income countries.

The evidence is highly concentrated on the outcome of transport infrastructure (especially roads), service and use. It is also concentrated in a specific region, East Asia and the Pacific, and urban populations.

Sectors with great development potential, such as waterways, are under‐examined, reflecting under‐investment.

**What is this map about?**

Transport interventions can play a key role in the achievement of many of the United Nations sustainable development goals (SDGs). This EGM contains the evidence base for all forms of transport: roads, bridges and paths, railways and trams, sea, ports and inland waterways, and civil aviation. For each part of the transport sector, the interventions are divided into infrastructure, incentives and institutions.

**What studies are included?**

Eligible studies had to be studies of the effects of a transport intervention, which is either the transport infrastructure or service itself, or transport‐related incentives or institutions.

Studies had to be impact evaluations designed to determine effects, including regression analysis. Before versus after, ex‐ante studies, and modelling studies without an empirical application are not included.

The EGM contains 466 studies, of which 34 are systematic reviews (SRs).

**What is the aim of this EGM?**

The aim of this EGM is to identify, map and describe existing evidence from studies reporting the quantitative effects of transport sector interventions related to all means of transport: roads, rail, trams and monorail, ports, shipping and inland waterways, and air transport.

**What are the main findings of this EGM?**

The studies are concentrated by sector and by outcome. The majority of the studies are in the intervention category of roads, bridges and paths, being mainly about roads. Of the three subcategories—infrastructure, incentives, and institutions—infrastructure is the most studied.

There is a moderate number of studies on railways, but the large majority of these are from East Asia, notably China. There are few studies on the other two intervention categories: sea and inland waterways, and air.

The studies follow the infrastructure. The large number of Chinese rail studies reflects the rapid growth in the Chinese railway system. The lack of studies of inland waterways in Africa reflects the lack of investment in this mode of transport.

The most frequently reported outcomes relate to transport use, such as mode of transport and travel time. This is followed by health and education, and economic development outcomes. Other outcomes are environment, equity and culture.

There are very few studies of known adverse effects like displacement.

Transport studies are under‐reviewed. Typically, 20%–30% of studies in an EGM are SRs. In this transport map, however, reviews make up only 34 studies out of 466, accounting for a 7% share. Moreover, the majority of the included reviews have methodological weaknesses, such as failure to conduct meta‐analysis and to assess risk of bias.

**What do the findings of this map mean?**

The map points to a clear research agenda. A first step would be to review the included SRs. Based on this analysis, and that of the map, consultation with stakeholders can determine research priorities for reviews and primary studies.

Since these studies contribute to the global public good of building the evidence base, this process is best done in a coordinated manner.

**How up‐to‐date is this EGM?**

The authors searched for studies published up to May 2020.

## 1 BACKGROUND

### 1.1 Introduction

*The problem, condition, or issue*.

#### 1.1.1 The condition

Context: *In the Footsteps of Mr Kurtz* Michela Wrong describes walking down the overgrown disused railway which years before had been part of a network linking DRC's copper mines to ports in Angola and South Africa. Despite new investments in the last decade—the Benguela Railway link from DRC to Angola re‐opened in 2018 after being closed for 34 years^1^—Africa's rail system is small compared to that in other parts of the world, and a substantial part of what there is not used (Bullock, 2009). The poor state of railway transport in Africa—and the unrealised potential of inland waterways—puts excess pressure on the fragile road transport system, so that transport costs—which are increased by uncompetitive practices—are a break on African development. Whilst much of Africa is an extreme case, inadequate transport infrastructure is an issue across much of the developing world.

There are great disparities in the quantity and quality of infrastructure. European countries such as Denmark, Germany, Switzerland, and the UK have close to 200 km of road per 100 km^2^, and the Netherlands over 300 km per 100 km^2^. By contrast, Kenya and Indonesia have <30, Laos and Morocco <20, Tanzania and Bolivia <10, and Mauritania only 1 km per 100 km^2^.^2^ As these figures show, there is a significant backlog of transport infrastructure investment in both rural and urban areas, especially in sub‐Saharan Africa (Foster & Briceño‐Garmendia, 2010). The situation is often exacerbated by weak governance and an inadequate regulatory framework with poor enforcement which lead to high costs and defective construction.

The wellbeing of many poor people is constrained by lack of transport, which is called “transport poverty”. Lucas et al. (2016) suggest that up to 90% of the world's population are transport poor which is defined as meeting at least one of the following criteria: (1) lack of available suitable transport, (2) lack of transport to necessary destinations, (3) cost of necessary transport puts household below the income poverty line, (4) excessive travel time, or (5) travel conditions which are unsafe or unhealthy.

Benefits of better transport: Better transport policies, infrastructure, and services are widely believed to be important to boost sustainable, inclusive growth in low‐ and middle‐income countries in other regions (Berg et al., 2017; Abdul Quium, 2019; Simon, 2002). Transport allows people to reach jobs, education, markets, social services and engage in social and political life. Sustaining rapid economic and social development in low‐and middle‐income countries presents a range of challenges for the transport system, a central one being to provide the capacity to accommodate increased volumes of passenger and freight traffic (Simon, 2002).

Cheap, efficient, adequate, safe, and sustainable transport services support agricultural and industrial production, inter‐ and intra‐county trade, regional integration, tourism, and social and administrative services that are key to national and regional development. Improved transport can affect:

- Production: Transport investments can transform economies by supporting structural change, notably the shift of the population from agriculture to manufacturing and services (e.g., Calderon, 2009; Kodongo & Ojah, 2016). A study of rural roads in Bangladesh found they reduced poverty through higher agricultural production, lower input and transportation costs, and higher agricultural output prices at local village markets, as well as increasing secondary school enrolment (Khandker et al., 2009). Incorporating transaction costs into a computable general equilibrium model of Uganda, Gollin, and Rogerson (2010) show that better infrastructure will stimulate agricultural production through higher farmgate prices.
- Consumption and prices: Better transport can make commodities more easily available and affordable. For example, the expansion of railways across India from the 1850s enabled market integration, which reduced prices of basic commodities such as salt.^3^ Transport‐induces changes in location of production and habitation (i.e., changes in land use) and so will affect land values. Deng et al. (2008) show that the increasing density of highways in China is a significant factor driving urban land expansion. Chalermpong (2007) estimates an elasticity of residential property prices with respect to distance from rail transit stations of −0.09.
- Access to services: Many studies show that long travel times, lack of transport services and high transportation costs are barriers to use of health services; for example. the systematic review (SR) by Kyei‐Nimakoh et al. (2017) of 160 studies of barriers to obstetric care in sub‐Saharan Africa.

These benefits are more fully elaborated in the theory of change below.

These benefits may not be realised, or be partly undermined, by the political economy context and the governance framework (Flyvbjerg, 2005; Klopp, 2012; and Alexeeva et al., 2008). Corruption and restrictive practices drive up costs, and public private partnerships often end up costing more than planned (Fatokun et al., 2015; Guasch et al., 2014). Transport costs are high in sub‐Saharan Africa even when the road infrastructure is adequate, due to a of lack of competition. Such considerations are an important part of the overall policy framework (Hine & Starkey, 2014), but beyond the scope of this map, which is concerned with quantitative studies of effectiveness, that is, the difference transport makes to outcomes of interest.

It is thus argued that better transport is a key component to achieving several sustainable development goals (SDGs): “There are a number of SDG targets directly linked to transport, including SDG 3 on health (increased road safety), SDG 7 on energy, SDG 8 on decent work and economic growth, SDG 9 on resilient infrastructure, SDG 11 on sustainable cities (access to transport and expanded public transport), SDG 12 on sustainable consumption and production (ending fossil fuel subsidies) and SDG 14 on oceans, seas and marine resources. In addition, sustainable transport will enable the implementation of nearly all the SDGs through inter‐linkage impacts. Access to sustainable transport for all should be at the forefront, including for vulnerable groups such as women, children, persons with disabilities and the elderly”.^4^

However, the presence and extent of these benefits depend on context: there is a great difference between those living in remote rural areas with little contact with the outside world and residents of a slum next to a highway in a rapidly growing city. How they interact with and can benefit from transport policies, of course, varies greatly. The impact of transport also depends on factors, such as employment opportunities, access to markets and distribution of health and education facilities, and other factors that may affect the use of all of these. The map must capture this full range of relevant interventions and possible policies, as well as the possible harms which may arise from transport.

Time frame: The time frame within which effects are realised varies. The adverse effects of displacement can happen almost immediately. Access to services can be relatively fast, though may depend on the development of transport services. Market opportunities and so growth may take longer to be realised, as will possible cultural effects.

Possible adverse consequences of infrastructure investments: Transport can bring disadvantages to some: displacement to make way for construction, poor road safety, higher land prices, spreading air pollution and disease, reduced accessibility on foot, moving access to jobs and goods further away, and adverse cultural effects.

Whilst transport infrastructure and services generally improve access to social services, they may have adverse effects on both health and education through the role of transport in spreading disease (the Black Death, HIV/AIDS in Africa in the 1980s and 1990s, and COVID‐19 in 2020—see, e.g., Apostolopoulos & Sonmez, 2006), accidents, and a busy road through a village stopping parents sending young children to school (Jeyaranjan et al., 2010). Over 80% of road traffic deaths are in developing countries (WHO, 2018).

Some of these factors are not captured in most analyses, so there is a risk that, if adverse effects are not measured, then the cost‐effectiveness of transport investments is overstated and they may not produce the full range of expected benefits, hence the importance of the regulatory framework. Understanding how transport policies can produce growth‐inducing effects and have social benefits, whilst considering possible adverse effects can guide setting priorities in the strategic use of scarce resources, and setting the regulatory framework for, transport investments. The challenge for transport development is thus to realise the benefits whilst minimising the adverse consequences.

### 1.2 The intervention

The intervention is the transport system itself and any intervention aiming to construct, improve, maintain or affect the use of that transport. The main categories in the map are modes of transport: roads, railways, trams and monorail, ports, shipping and inland waterways, and air transport. For each mode of transport, the subcategories are infrastructure, incentives, and institutions (including regulations). A single study may cut across the various subcategories for a specific form of infrastructure, or less commonly analyse multiple forms of transport (or the connections between them).

The subcategories are the three policies that contribute to improving transport networks: (i) infrastructure investments, (ii) price instruments (which we label more broadly as incentives), and (iii) regulations (Berg et al., 2016). More specifically:

- Infrastructure entails building new transport infrastructure (e.g., roads, railways, ports, or airports), upgrading existing links and technology, or improving transport services.
- Incentives include subsidies or taxes to influence mode choice and transport behaviour (e.g., student fare reductions, tolls, parking fares, fuel taxes, and clean transport subsidies).
- Institutions (regulations) include rules to directly reduce emissions, such as fuel emission standards or driving restrictions or to organise the transport sector (e.g., freight, taxis, or buses) or standards for the construction of infrastructure.

Some policy interventions may affect supply, such as infrastructure, whereas others target demand, such as subsidies for transport.

### 1.3 Why it is important to develop this EGM

Although there is no separate SDG for transport, of the 17 SDGs, seven goals (SDG 2, 3, 7, 9, 11, 12 and 13) include one or more targets that address transport, both rural and urban; and four (SDG 2, 3, 9, and 11) make specific reference to transport and infrastructure (United Nations 2016). According to the Institute of Transportation and Development Policy, “this elevation of transport in SDGs recognises it as a key tool in reducing emissions, improving equity, and reducing poverty”. Analysis of these goals identifies the following key aspects of transport in the SDGs: access (urban, rural, affordable for all), road safety, fuel type/efficiency; quality, reliability, resilient, and sustainable infrastructure; regional and trans‐border transport; sustainable urban transport for all; reduce vehicle emissions/air pollution in cities; reform fossil‐fuel subsidies; rural/urban logistics, supply chain efficiency; and mitigation and adaptation of climate change.

The literature on the impact of transport policies covers a variety of interventions and outcomes at different levels, such as micro, meso and macro. Due to the wide variety of interventions, mechanisms and outcomes, a simple way to formalise the impact of transport policies is to how these policies affect the welfare of individuals or groups, improve regulation and infrastructure, would be quite useful. At the same time, as explained above, the expansion of transport in LMICs has brought out both positive and negative effects.

The literature on the impact of transport policies covers a variety of interventions and outcomes at different levels, such as micro, meso and macro. Due to the wide variety of interventions, mechanisms and outcomes, a simple way to formalise the impact of transport policies is to how these policies affect the welfare of individuals or groups, improve regulation and infrastructure, would be quite useful. At the same time, as explained above, the expansion of transport in LMICs may have both positive and negative effects.

The purpose of this map is to document all relevant studies, from all sectors, which analyse the effects of transport interventions. The nearest studies to what we will do is the ADB review of transport impact evaluations by Raitzer et al. (2019) and the review of transport corridors by Roberts et al. (2019). However, the first of these review was not systematic and more restricted to analysis by economists, and the second is restricted to large infrastructure. We have a broader disciplinary coverage than the first and broader topic coverage than the secod.

#### 1.3.1 Existing EGMs and/or relevant SRs

A map of evidence maps conducted in low‐ and middle‐income countries identified no EGM conducted around transportation (Phillips et al., 2017). The lack of such a map was the rationale for undertaking this map. There is an on‐going global map of road safety (Mohan et al., 2020).

Table 1 lists some reviews of transport sector interventions. These are illustrative of the sort of topics, which may be covered; they have not been screened to determine whether they include primary studies from low and middle‐income countries.

**Table 1:** Systematic review of transport systems

Interventions	Roads, cycle paths and pavements/walkways	Railways	Shipping and waterways
Investments and maintenance	Egan et al. (2011); New roads and human health	Havârneanu et al. (2015); A systematic review of the literature on safety measures to prevent railway suicides and trespassing accidents	
	Benítez‐López (2010); The impacts of roads and other infrastructure on mammal and bird populations: a meta‐analysis	Bastiaanssen et al. (2020); Does transport help people to gain employment? A systematic review and meta‐analysis of the empirical evidence	
	Cavil et al. (2008); Economic analyses of transport infrastructure and policies including health effects related to cycling and walking	Kasraian et al. (2016)	
	Hine et al. (2015); The poverty reduction impact of rural roads: a systematic review; and	Long‐term impacts of transport infrastructure networks on land‐use change: an international review of empirical studies	
	Hine et al. (2019). Evidence on impact of rural roads on poverty and economic development		
Information and incentives	Ogilvie et al. (2004); Promoting walking and cycling as an alternative to using cars: systematic review		
Policy and regulatory environment	Heath et al. (2006) The effectiveness of urban design and land use and transport policies and practices to increase physical activity: a systematic review		Vieira et al. (2014); Governance, governance models and port performance: a systematic review

*Note*: Air transport excluded as no relevant reviews were found. A reviewer mentioned an on‐going review of Air Transport in Low‐ and Middle‐Income Countries by Foster and Bofinger, which but we have not located it (there are several papers by Bofinger, but no review).

## 2 OBJECTIVES

### 2.1 Objectives

The EGM aims to identify, map and describe existing evidence on the effects of transport sector interventions related to all means of transport (roads, railways, trams and monorail, ports, shipping and inland waterways, and air transport) in low‐ and middle‐income countries. For each sector, these interventions are classified as infrastructure, incentives, and the institutional framework (including regulations). The primary outcomes of this EGM include transport infrastructure, economic impact, health and education, environmental, economic, and equity outcomes.

The objectives of this EGM map are to:

a) Develop a clear framework of interventions and outcomes related to the effects of transport in low‐and middle‐income countries
b) Map available SRs and primary studies of the social and economic effects of interventions aimed at improving transportation in low‐ and middle‐income countries in this framework, with an overview provided in a summary report.
c) Provide database entries of included studies, which summarise the intervention, context, study design, and main findings.

The map has been produced in accordance with the Campbell Collaboration Guidance for the productions of evidence and gap maps (EGMs; White et al., 2020). The search of the evidence started from April 2020 and the analysis of the map started in October 2020 and added additional studies in September 2021.

#### 2.1.1 Snapshot of transport EGM

**Figure 1:**
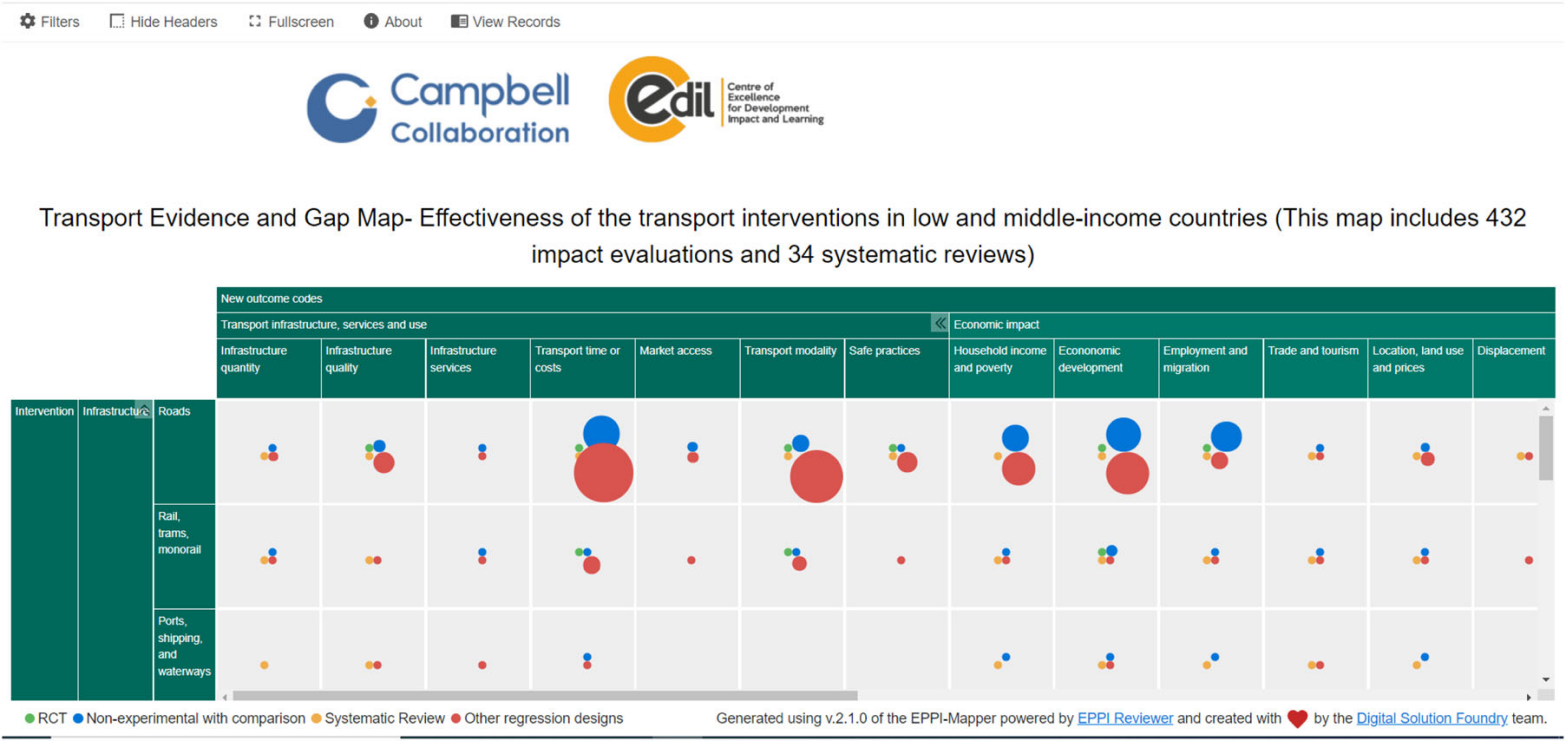
Snapshot of the transport map

The intervention and the outcomes are the primary dimensions of this map. The online map also shows the secondary dimension (filters) such as region, population, study methods, etc. (see Figure 1). The bubbles indicate the study design, green colour denotes other regression designs, blue denotes nonexperimental with a comparison group, and red denotes randomised controlled trials (RCTs). SRs are shown by brown bubbles. The size of the bubbles indicates the volume of the evidence in that cell.

## 3 METHODS

### 3.1 EGM: Definition and purpose

#### 3.1.1 Defining EGMs

While SRs aim to identify, assess and summarise research findings from studies on a (narrow) research question, the objective of EGMs is to provide a picture of the coverage of existing research literature on a given topic. As such, EGMs have a broader scope than SRs, and SRs go further than EGMs in processing the contents of the identified research. Another important difference between EGMs and SRs is how they are disseminated. SRs are disseminated as research reports or journal articles, where the answer to the research question is the key issue for readers. EGMs can also be disseminated as a report or an article, but the more user‐friendly EGMs display its results in an interactive matrix. Identified studies are plotted in the matrix, so that the user can find evidence, or lack thereof, for his or her particular topic of interest, at a glance. EGMs are global public goods that attempt to democratise high quality research evidence for policy makers, practitioners, and public and research funders.

The EGM presented here includes evidence from impact evaluations and SRs. Any single study may appear in multiple cells if it covers more than one category or subcategory for either intervention or outcome.

##### 3.1.1.1 Type of population (as applicable)

The target population for this EGM is populations living in low‐ and middle‐income countries. Rural/urban and global regions by World Bank classification are included as population subgroups. These subgroups are added to the map as filters.

##### 3.1.1.2 Types of interventions/problem

The EGM includes intervention categories, which are each mode of transport such as roads, paths, cycle lanes, bridges, railways, ports, shipping and inland waterways, and air transport, and the subcategories are infrastructure, information and incentives, and institutions. Table 2 shows the resulting set of intervention categories.

Since the subcategory labels are the same across all categories it is possible in the visual EGM to swap the categories and subcategories. The authors will present the map in both layouts.

##### 3.1.1.3 Types of outcome measures

The outcomes are listed in outcome domains (see Table 3). Each domain has a number of subdomains. The map covers positive and adverse outcomes, with outcomes being broadly defined so as to capture unintended outcomes. The selection of outcomes is informed by the theory of change which is presented below (Figure 3).

**Table 2:** Intervention categories and subcategories

Category	Subcategories	Examples
Road, paths, and footbridges	Infrastructure	Construction and upgrading of roads, and highways
		Infrastructure maintenance
	Incentives	Road pricing and tolls
		Subsidies and taxes
	Institutions (including regulations)	Road legislation and agencies
		Vehicle and driving regulations
		Public‐private partnership (PPP)
Rail and trams	Infrastructure	Construction and upgrading
		Maintenance
	Incentives	Pricing structure
		Subsidies to rail operators
	Institutions (including regulations)	Regulatory framework
		Public‐private partnership (PPP)
		Nationalisation/privatisation
Ports, shipping, and waterways	Infrastructure	Port and inland waterway construction and rehabilitation including modernisation
		Maintenance
	Incentives	Tolls and other charges
		Taxes and subsidies
	Institutions (including regulations)	Port authorities
Civil aviation	Infrastructure	Airports
	Incentives	Taxes and subsidies
	Institutions (including regulations)	Airport authorities

**Table 3:** EGM outcomes

Domain	Subdomain
Transport infrastructure, services, and use	Infrastructure quantity
	Infrastructure quality (inc. safety assessment)
	Infrastructure services
	Transport time or costs (inc. congestion and VOC)
	Market access
	Transport modality (inc. car ownership)
	Safe practices
Economic Impact	Household income and poverty
	Economic Development
	Employment and migration
	Trade and tourism
	Location (land use) and prices
	Displacement
Health and education	Access to health facilities
	Health outcomes
	Access to education facilities
	Education outcomes
Culture	Values, language, and social cohesion
	Cultural heritage
	Cultural diversity
Environment	Air quality
	Noise pollution
	Habitat destruction
Economic and equity analysis	Cost‐effectiveness or CBA
	Gender equity
	Transport equity^a^

^a^ Transportation equity or justice usually refers to the fairness with which the impacts of transportation such as benefits and costs are distributed. Horizontal equity, also called fairness and egalitarianism, is concerned with the distribution of impacts between individuals and groups considered equal in ability and need; vertical equity is concerned with the distribution of impacts between individuals and groups that differ in abilities and needs, for example by income or social class (also called social justice, environmental justice and social inclusion) or in transportation ability and need otherwise known as universal design (Litman, 2018)

#### 3.1.2 Criteria for including and excluding studies

##### 3.1.2.1 Types of study designs

There are many policy‐relevant areas of research on transport, including barriers to access, costs and governance arrangements. Qualitative data and studies can play an important role on complementing impact evaluations; see White (2011) on mixed methods impact evaluations in infrastructure. However, this transport map is a map of effectiveness studies which measure the change in outcomes attributable to the interventions, and so excludes qualitative studies. The rationale is the comparative lack of measures of impact on outcomes of interest using impact evaluation methods. But there is a growing literature. Making this literature discoverable and accessible is the main contribution of this map.

The map is timely because the number of impact evaluations has been growing across development sectors. By impact evaluations we mean studies which assess the difference an intervention makes to outcomes, employing a technique which handles the possible endogeneity of exposure to the intervention (though the extent to which this is done satisfactorily varies by method). This endogeneity is at the heart of discussions on transport and development. In the *Handbook of Transport and Development* (in which the cases are mostly from developed countries), the authors state in the introduction that “Often it seems that development follows the transport infrastructure… But the causality is rarely in one direction and often the development form helps shape the transport infrastructure investments” (Hickman et al., 2015: 3).

##### 3.1.2.2 Types of evidence

This EGM includes ongoing and completed impact evaluations and SRs of the effectiveness of transport sector interventions. This is a map of effectiveness studies. The impact evaluations include:

- Experimental designs: RCTs and natural experiments.
- Non‐experimental designs: (i) quasi‐experimental designs using statistical methods to create a comparison group such as propensity score matching and regression discontinuity, (ii) regression‐based designs such as instrumental variables and Heckman sample selection models; and (iii) other studies with a comparison group. Before versus after studies with no comparison group are not included.
- Regression designs which control for confounding variables.

We do not include before versus after studies, ex ante impact estimates including cost–benefit analysis, or modelling studies without an empirical application.

##### 3.1.2.3 Types of settings (as applicable)

All included impact evaluations must have been conducted in low‐ and middle‐income countries (LMICs) as defined by the World Bank. SRs containing evidence only from high‐income countries are excluded.

Status of studies

We searched for and included completed and on‐going studies. We have restricted our search to English language. We did not exclude any studies‐based on publication status or publication date.

##### 3.1.2.4 Search methods and sources

**Figure 2:**
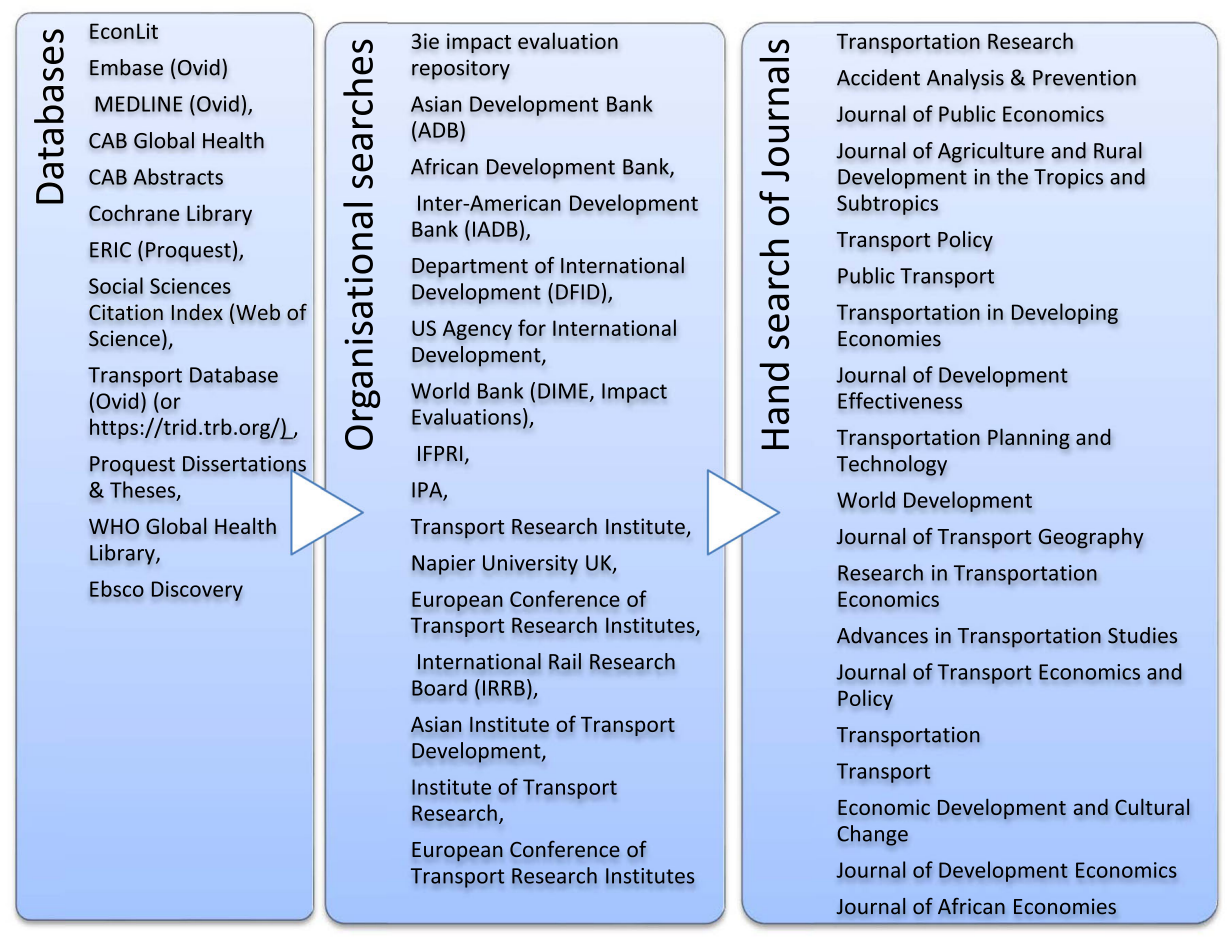
Search sources

The search strategy included 12 databases, and more than 20 relevant websites, and a hand search of more than 20 journals (see Figure 2). The authors also included grey literature from Google as well as the listed websites. We conducted bibliographic back‐referencing of reference lists of all included SRs to identify additional primary studies and SRs.

In addition, we identified the developing country studies from the ongoing map of road safety interventions (Mohan et al., 2020). All screening was done independently by two people (SM, NDC) with a third‐party arbitrator in case of disagreement (HW).

##### 3.1.2.5 EGM Protocol

The EGM protocol was published on 21, January 2021 (Malhotra et al., 2021).

### 3.2 Stakeholder engagement

The choice of transport as a map was based on the map of maps (Phillips et al., 2017) which identified a gap in this area, and was seen as a priority by the funder, that is the UK Foreign, Commonwealth, and Development Office (FCDO).

We have engaged stakeholders in developing the evidence matrix at the various organisation that works on transport sector interventions. These include:

- TERI University (Department of Civil Engineering)
- IIT‐Delhi,
- and Independent Council for Road Safety International (ICORSI).

Earlier versions of the map have been presented at ADB in Manila (September 2019), in the What Works Global Summit 2020 (October 2020), and in the Campbell Collaboration webinar series (December 2020). A draft of the map report was shared with ADB, African Development Bank, the Millennium Challenge Corporation, and FCDO.

### 3.3 Dimensions

#### 3.3.1 Scope

The scope of this map covers (1) types of transport; (2) the policies and other actions to promote transport‐related development; (3) the outcomes of interest; (4) the population of interest; and (5) eligible study designs.

The map included the interventions related to all kinds of transport: rail/tram, road and on foot or bike by land, both inland waterways and international maritime transport, and air.

#### 3.3.2 Conceptual framework

**Figure 3:**
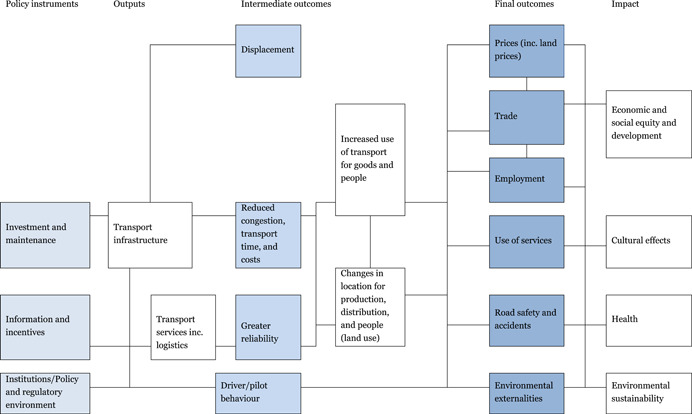
General theory of change for transport interventions

Several sources present theories of change figured for transport interventions (e.g., Berg et al., 2017; Abdul Quium, 2019; Raitzer et al., 2019). Our theory of change, shown in Figure 3, draws on each of these to give a high‐level representation that applies to all our included modes of transport. The framework identifies common causal pathways for the different modes of transport, meaning that there are likely to be common lessons across sectors that may get overlooked by researchers and policymakers specialised in just one sector.

The theory of change shows the causal chains through which inputs are turned into outputs, intermediate and final outcomes, and higher‐order welfare effects (impact). On the left of the figure are the intervention areas of investment and maintenance, information and incentives, and the institutional framework (policies and regulations). These effects are mediated by the political economy context and governance framework.

The availability of transport infrastructure and services affects the mediating variables through reduced travel time and greater reliability which drive location decisions for production and people, and so transport and commuting. These in turn, and together, affect a whole range of final outcomes, some of which further interact prices, internal and external trade, employment, use of services, road safety and accidents, and a range of positive and negative environmental externalities. The time taken to realise these different outcomes will vary, with some being realised immediately and others taking some years.

These effects on outcomes lead onto the changes in higher‐order welfare effects (impacts) under the broad headings of:

- Economic and social equity and development: Effects on both economic development through trade, productivity and growth, and social development in various forms through better access. Adverse effects on displaced populations who lose their land or livelihood are also captured here. Transport planning may mean that transport makes life harder for the poor note easier if the way in which they travel is marginalised, such as roads without pedestrian access.
- Cultural effects: The positive and negative consequences of increased mobility within and between nations. The increased mobility of the population may have effects on the cultural beliefs, values, customs, and norms. An example is a cultural heterogeneity resulting from migration to urban areas that can result in the loss of traditional values.
- Health: Health is separated as there are many channels through which transport can affect health, both positive (access to health services, higher income, availability of more diversified diet, etc.) and negative (road traffic injuries, air pollution, and spreading disease).
- Sustainability: Transport can have adverse effects on the environment, through the impact on land use and local flora and fauna. Congestion is a growing problem, contributing to air pollution from increased traffic volumes.

This framework is used to define the categories of interventions and the outcomes along the causal chain to be shown in the map.

### 3.4 Description of intervention

Table 1 above lists the intervention categories and subcategories.

### 3.5 Description of population/geographic location/outcome categories

Included studies were those that include population from low‐ and middle‐income countries and reported the transport sector intervention and on the main six outcomes.

The six main outcomes, which follow from the theory of change, are:

- Transport infrastructure, services, and use
- Economic Impact
- Health and education
- Culture
- Environment
- Economic and equity analysis.

The outcomes categories and subcategories are given in Table 2.

### 3.6 Analysis and presentation

#### 3.6.1 Unit of analyses

In the EGM, where multiple papers exist on the same study only one is included if they are the same (e.g., working paper and a published version), the most recent open access version included in the EGM. Where different papers from the same study report different outcomes then all such papers will be included.

##### Presentation

This EGM has two primary dimensions: intervention as rows and outcomes as columns. The map displays the interventions (road, rail and trams, ports, shipping and waterways, and civil aviation), subcategory (infrastructure, incentives, institutions (including regulations) against outcomes for each mode of transport. In the online map we used secondary dimensions:

1. Study design
2. Population (Rural, Urban, and Both)
3. Region: East Asia & Pacific, Latin America & Caribbean, Middle East & North Africa, South Asia, sub‐Saharan Africa, Europe & Central Asia.

For this map, we present two forms of visualisation of the evidence with the categories and subcategories swapped around.

## 4 DATA COLLECTION AND ANALYSIS

### 4.1 Screening and study selection

The screening for inclusion/exclusion of studies was undertaken in two stages using EPPI reviewer 4. The first stage involved title and abstract screening and the second involved the screening of the full text. Both stages of screening were done by two independent researchers (S. M. and N. D. C.) against the predefined inclusion criteria for the map, with a third‐party arbitrator in case of disagreement (H. W.).

### 4.2 Data extraction and management

For impact evaluation and SRs, we used a standardised data extraction form (Annexure 1) to extract descriptive data from all the studies that met our inclusion criteria. Data extraction from each study included context/geographical information, population, study design and method, intervention types and outcomes type, and subcategory. Two researchers (S. M. and N. D. C.) conducted the data extraction for each study. Both coders were trained on the tool before starting. Disagreements were resolved through discussion with a third reviewer consulted as needed (H. W.).

### 4.3 Tools for assessing the study quality of included reviews

All SRs were appraised for quality using the AMSTAR2 tool. Critical appraisal assessment was completed by two reviewers (S. M. and N. D. C.).

The 16 items in AMSTAR2 cover:

1. PICOS in inclusion criteria,
2. Ex‐ante protocol,
3. Rationale for included study designs,
4. Comprehensive literature search,
5. Duplicate screening,
6. Duplicate data extraction,
7. List of excluded studies with justification,
8. Adequate description of included studies,
9. Adequate risk of bias assessment,
10. Report sources of funding,
11. Appropriate use of meta‐analysis,
12. Risk of bias assessment for meta‐analysis,
13. Allowance for risk of bias in discussing findings,
14. Discussion and analysis of heterogeneity,
15. Assessment of publication bias,
16. Report and potential source of conflicts of interest.

Seven domains can critically affect the validity of a review and its conclusions (critical items 2, 4, 7, 9, 11, 13, and 15). The study's overall confidence ratings of the quality are high if there is no more than one noncritical weakness, medium if there is no critical weakness but more than one noncritical weakness, and low if there are one or more critical weaknesses.

We did not critically appraise the quality of the included impact evaluations but collected data on study design.

## 5 RESULTS

### 5.1 Description of studies

#### 5.1.1 Results of the search

The database search identified 5325 of which 211 were duplicates, leaving 5191 studies for the title and abstract screening. Of these, 458 studies were screened for full text. We have excluded 146 studies at the full‐text screening stage.

**Figure 4:**
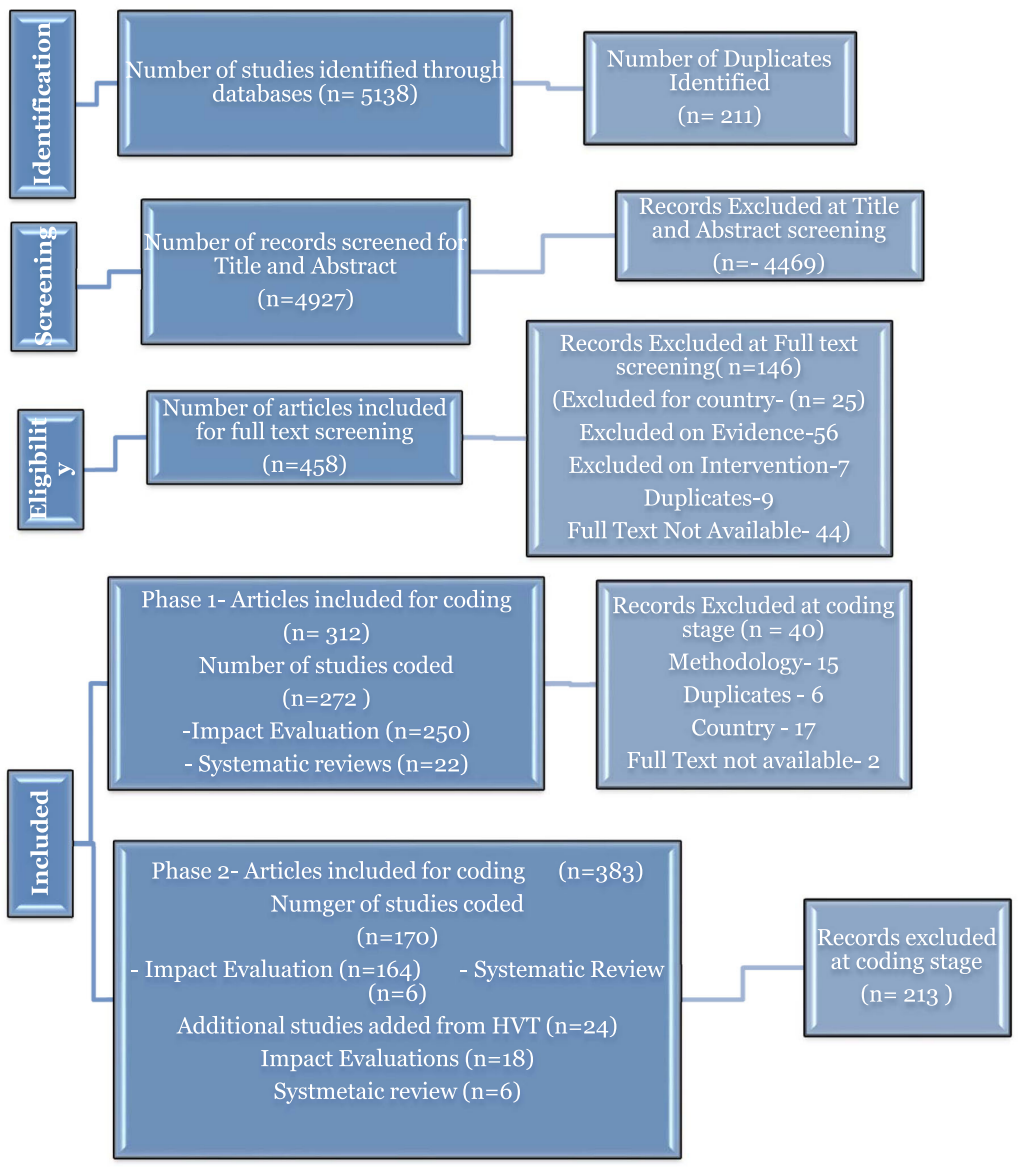
PRISMA flow chart

Finally, we have included 312 studies for coding. We excluded 40 studies due to study methodology, location, and intervention at the coding stage. This left 272 included studies of which 250 are impact evaluations and 22 SRs. This is identified as Phase 1 in the PRISMA diagram (Figure 4).

Phase 2 of the search is based on the grey literature search of the various organisational websites, hand searches of journals, and bibliographic searches. As a result of these searches, we included an additional 383 studies for coding. Of these 45 studies were from the grey literature search, 61 studies from back referencing, and 149 studies included hand searches from 19 journals. A further 128 studies were identified from the road safety intervention EGM (Mohan et al., 2020). The majority of these studies (213) were excluded on closer examination at the coding stage, resulting in 170 additional studies on the map (see Figure 4). We also added 24 additional studies from the search to High Volume Transport Applied Research Programme (HVT) website.

**Figure 5:**
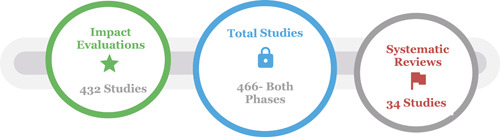
Overview of included studies

As a result of both phases, we have included 466 studies that met our inclusion criteria: 432 impact evaluations and 34 SRs (Figure 5).

#### 5.1.2 Overview—Interventions and outcomes

In the aggregate map (Table 3a) we present the aggregate map by intervention category (mode of transport) and outcomes. The most striking finding is that the dominance of studies on roads, bridges, and paths (which are mainly roads). There is a reasonable number of studies on rails and trams (which, as shown below, are mostly from East Asia, mainly China). There are very few studies indeed on transport by air, sea, and inland waterways, reflecting in part the neglect of these as a transport system.

**Table 3a:** Aggregate map, number of studies by transport category and outcome category: total studies and systematic reviews (in brackets)

	Roads, bridges and paths	Rails and trams	Ports, sea and inland waterways	Civil aviation	Total
Transport infrastructure, services, and use	314 (18)	60 (2)	5 (1)	9 (2)	**351**
Economic impact	182 (8)	49 (6)	12 (4)	6 (4)	**218**
Health and education	135 (17)	9 (0)	2 (1)	3 (2)	**139**
Culture	6 (2)	2 (2)	1 (1)	1 (1)	**6**
Environment	58 (8)	12 (1)	1 (1)	2 (2)	**65**
Economic and equity analysis	36 (5)	8 (3)	2 (2)	1 (1)	**39**
Total	**408**	**82**	**14**	**14**	**466**

Waterways remain an underused means of transport in Africa and South Asia. The Ganges saw little growth in freight traffic from 1945 to 95, with modest growth thereafter. Freight on the Congo remained stagnant from 1945 to 2015. By contrast, traffic on the Yangtze increased more than fourfold over the same period (Wang et al., 2020). Of course, there is undoubtedly an endogeneity: economic development increases freight transport as well as better waterways facilitating economic development. Given the exogenous placement of waterways, analyzing their impact is a tractable problem, but there is no paper presenting this analysis. The closest study to this issue is that of Iimi et al., 2015 showing that access to ports substantially increases exports of cash crops such as coffee, tea, tobacco, and cotton in African countries.

The most commonly reported outcomes are access to transport, economic impact, and health and education in that order. Hence the most heavily evidenced cells—with over 100 studies per cell—are the three cells for these outcomes for roads, bridges and paths. Much of the map has few, or even no, entries, pointing to substantial evidence gaps. As already noted, these affect transport modes (air and water) and some outcomes, notably culture, but also economic and equity outcomes, environment and, health and education for all transport modes other than roads.

The distribution of the SRs follows roughly the same pattern as primary studies. Most reviews are about roads, with few on other means of transport. However, the main outcome is health—which is common for reviews as this is best established in health reviews. However, there are also a reasonable number of reviews on economic development. A useful follow‐on product from this map would be an overview of reviews contained in the map, one output of which would be to identify (in conjunction with additional analysis as to what is covered by the primary studies in the map) a list of potential topics for additional SRs.

Table 3b shows the aggregate map with the categories now shown as infrastructure, incentives, and institutions. This shows that the most well‐evidenced area is infrastructure across the main outcome categories already noted. There are a reasonable number of studies on institutions, mostly about transport use. There are the fewest on incentives, and again mostly on transport use.

**Table 3b:** Aggregate map, number of studies by intervention category and outcome category

	Infrastructure	Incentives	Institutions (including regulations)	Total
Transport infrastructure, services, and use	277	23	69	351
Economic impact	201	8	17	218
Health and education	119	3	24	139
Culture	5	0	1	6
Environment	45	2	22	65
Economic and equity analysis	31	0	11	39
Total	373	28	87	466

In all these tables the totals do not sum since a study may appear in more than one cell.

#### 5.1.3 Evidence base by intervention

**Figure 6:**
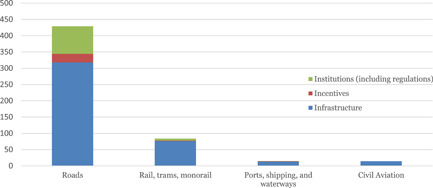
Results on the intervention categories and subcategories

Figure 6 shows the above findings graphically. The dominance of studies on roads is clear, as is the preponderance studies on infrastructure: 77% of the included studies are about infrastructure as an intervention, and the majority is about road infrastructure (79%). We find very few studies on ports and shipping or civil aviation.

**Figure 7:**
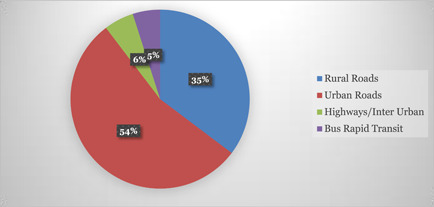
Studies as per interventions in different road categories

In road infrastructure, there is available evidence on the interventions on Urban Roads (242 studies), Rural Roads (157), Highways/Inter Urban (24 Studies) and Bus Rapid Transit (22 studies) (Figure 7).

**Figure 8:**
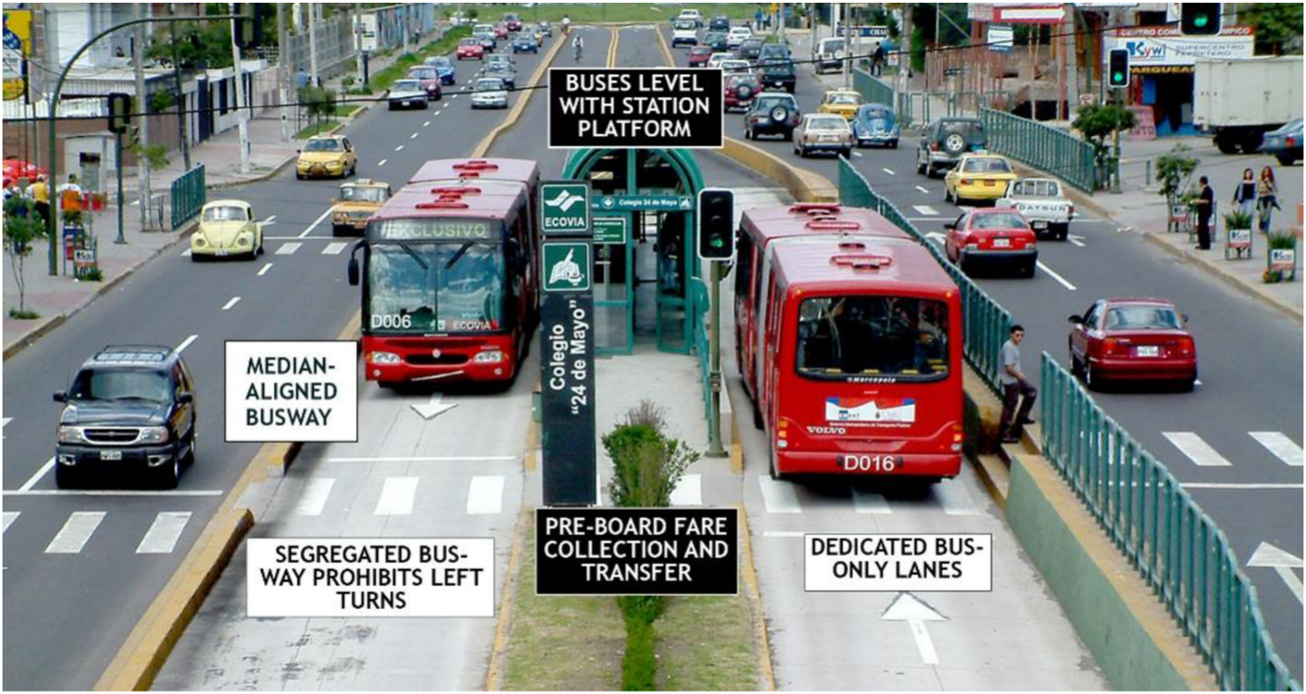
Bus rapid transit bus stop in Quito, Ecuador

In road infrastructure, there are many studies (22 studies) related to Bus Rapid Transit (BRT) systems. This is a high‐quality bus‐based transit system and provides dedicated lanes to buses. Figure 8 shows an example from Quito, Ecuador.

There are 79 studies in the map on rail, trams, and monorail. Sixty (79%) of these studies are from East Asia, mainly China. This focus on railway studies reflects the rapid growth of the Chinese railway system in recent years, both within and between cities. The number of cities with urban rail lines in use or under construction grew from 25 in 2008 to 63 in 2015, with the length of a line in use quadrupling from 803 to 3293 km (Lu et al., 2016). Under the 14th Five Year Plan (2021–2025) an additional 10,000 km of rail with be built.^5^

#### 5.1.4 Evidence base by outcome category and subcategory

**Figure 9:**
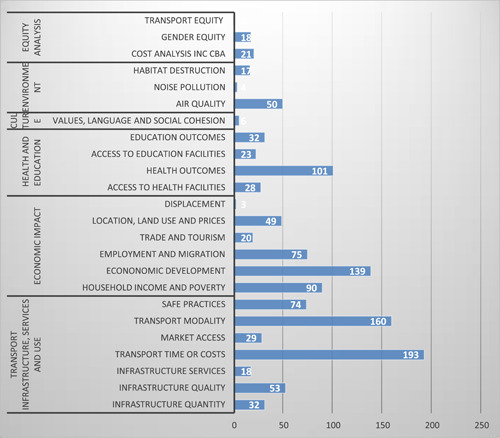
Result of the outcome categories and subcategories

The evidence base is largest for the outcomes related to transport time or cost and transport modality (Figure 9). There are also many studies reporting economic development which includes growth, firm and enterprise development, and agricultural production. Other well‐studied economic outcomes are household income and poverty, employment, and migration. Other outcomes with reasonable evidence are health outcomes, air quality, and road safety. As already noted, there are few studies of cultural effects and on the adverse outcomes of displacement and habitat loss.

#### 5.1.5 Secondary dimensions of the map

##### Study design

**Figure 10:**
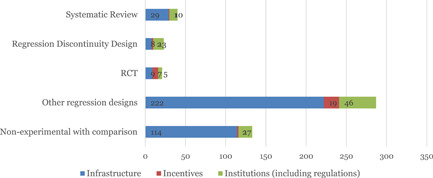
Number of studies as per the study design and interevention subcategories

Of the 466 studies on the map the most common design are regression studies (274 studies; Figure 10), and another 131 studies with nonexperimental designs with a comparison group. There are very few randomised controlled trials (20 studies).

SRs make up just 7% (34 out of 466) studies in the map. This is a low percentage compared to most other maps. For example, the disability map has 59 reviews out of a total of 166 studies, that is 36% (Saran et al., 2020). Transport is thus an under‐reviewed area. As proposed above, the map should be used to identify additional SRs which would be of interest to decision‐makers.

##### Region

**Figure 11:**
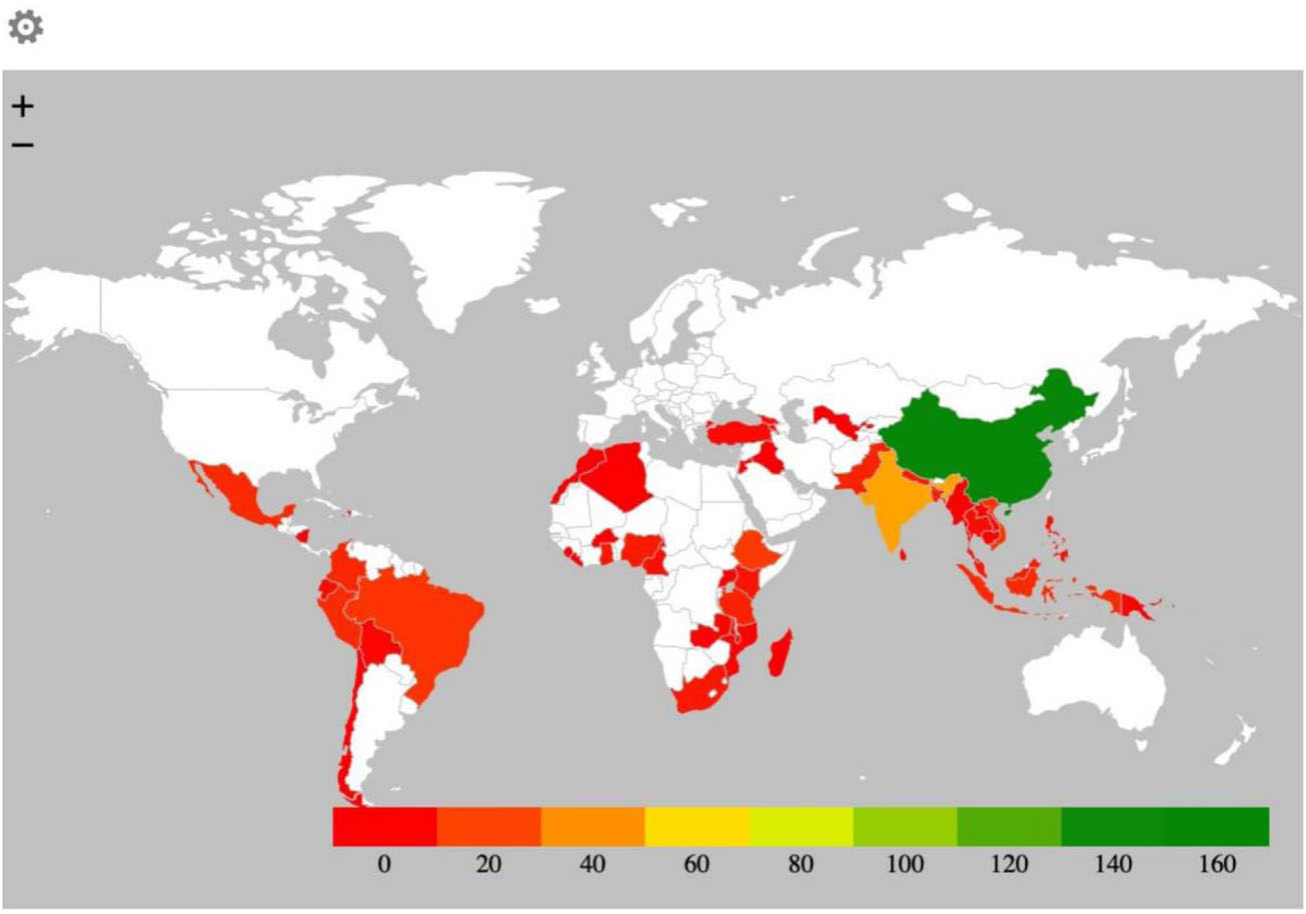
Geographical Heat Map of the studies included in EGM

The most well‐represented region in the map is East Asia and the Pacific (223 studies, 40%), which is more than double the share of the next most well‐represented region (sub‐Saharan Africa with 108 studies). Amongst East Asia and the Pacific countries, the majority of the studies are from China (141 studies, 31%; Figure 10). There are very few studies from Europe and Central Asia and the Middle East and North Africa. There are 79 studies from South Asia with the most studies from India (44 studies). The countries with the highest evidence concentration are China, mentioned above (141 studies), next is India (44 studies), and Ethiopia (16 studies) (Figure 11).

**Figure 12:**
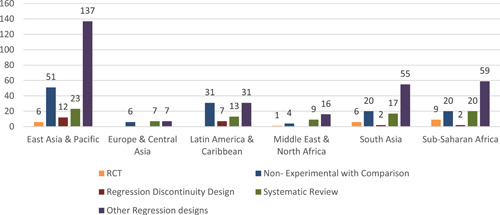
Number of studies by study design and by region

Region‐wise study design: In East Asia and Pacific the most used study designs are other—as for the whole map—is other regression design, followed by nonexperimental designs with a comparison group. The same pattern is seen in other regions except for Latin America and Caribbean, where comparison group designs are most common (Figure 12).

##### Population groups

**Figure 13:**
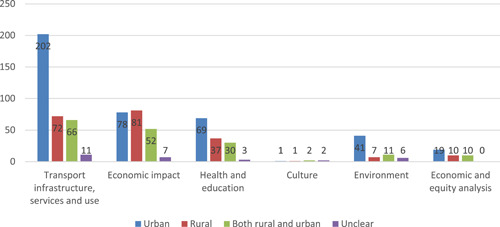
Distribution of urban and rural studies by outcome

The effects of transport were studied for the urban population in a just over half of the studies (56% of total 466 studies), compared to under quarter (21%) considering rural population, and 19% covering both rural and urban. Urban studies look disproportionately at transport use, whereas rural studies are more concerned with economic impact (Figure 13).

### 5.2 Status of included studies

There are 460 completed studies and 6 ongoing studies included in the map.

### 5.3 Quality appraisal in included reviews

There are 34 SRs. We critically appraised the quality of the reviews by using AMSTAR‐2. Among the included reviews, 85% of the SRs rated as low and 12% rated as medium confidence in study findings. Only one review was rated as high confidence.

**Figure 14:**
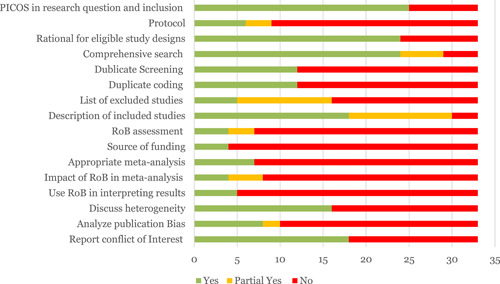
AMSTAR2 assessment

As shown in Figure 14, major limitations were the absence of risk of bias analysis, not undertaking meta‐analysis‐only 1 study executed a meta‐analysis, failure to use two screeners and coders (or at least a failure to report doing so), failure to have a protocol, and not declaring sources of funding. This assessment of the shortcomings in existing reviews reinforces the case for commissioning a new programme of reviews of transport studies.

## 6 DISCUSSION AND GAPS IN EVIDENCE

### 6.1 Summary of main results

This map has 466 studies, of which 34 are SRs.

**Figure 15:**
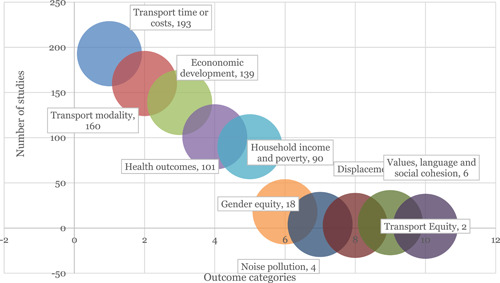
Evidence concentration and gaps in categories among outcome

The majority of the studies are about road‐related interventions and on infrastructure development. Most of the studies measured the impact of the intervention on transport cost and time and mode of transport used (Figure 15).

The East Asia and Pacific region accounts for the largest share of studies (40%), with most of these coming from China. Just over half of studies concern the urban population (56%).

Sectors other than roads are relatively neglected in the evidence base. Whilst there are a sizeable number of studies on railways, most of these are from one country (China). There is very little evidence on waterways, whose potential remains unrealised especially in sub‐Saharan Africa.

There is very little evidence on equity analysis and culture. It is possible that cultural effects are more studied in qualitative literature. Only six studies among 466 about culture outcomes and only four studies measured noise pollution. Only 18 studies measured the effects of transport on gender equity, 21 studies applying ex‐post cost‐benefit analysis, and two studies reported findings for transport equity.

The low ratio of reviews to primary studies makes this an under‐reviewed area. Moreover, most of the reviews have methodological shortcomings, such as a failure to conduct and use the risk of bias analysis and to undertake meta‐analysis where appropriate.

### 6.2 Areas of major gaps in the evidence

There are many blank cells in the intervention categories in civil aviation, and very few outcome categories related to culture. Most of the studies are concentrated on specific regions and countries. There is a need for more studies from Latin America & Caribbean, Europe & Central Asia, and the Middle East and North Africa. And there is a lack of experimental studies on transport sector intervention even in areas though studies of incentives may be possible.

We found notable gaps in the evidence related to the intervention on the ports, shipping and no evidence on waterways. The evidence is very much concentrated on infrastructure development and use, economic impact, and health outcomes. There is a striking gap in studies that focus on the effects of transport on cultural heritage and diversity. There is a lack of evidence on outcomes such as noise pollution, values, language, and social cohesion, transport equity, and displacement

There is very little evidence on environmental outcomes such as air quality. There are also few studies on equity issues such as gender equity.

The extent to which these gaps need to be filled depends on whether these are priority areas for policy‐makers.

### 6.3 Potential biases in the mapping process

In terms of biases, in the selection process, we have selected evidence available in the English language.

### 6.4 Limitations of the EGM

i. Eligible studies were restricted to those published in English.
ii. Searching the grey literature is challenging, and consequently, some eligible studies may have been missed.

### 6.5 Stakeholder engagement throughout the EGM process

We have engaged stakeholders on the evidence matrix at the various organisation that work on transport sector interventions. These include TERI University, Department of Civil Engineering, IIT‐Delhi, and Independent Council for Road Safety International (ICORSI).

The draft report was shared with ADB, and African Development Bank as well FCDO (DFID) and MCC.

## 7 AUTHORS' CONCLUSIONS

The mapping exercise has two goals:

- Facilitate access to, and use of, research on the effectiveness of transport interventions through the online interactive visualisation of the map and accompanying list of references; and
- Identify priority areas for SRs and impact evaluations for transport.

### 7.1 Implications for research, practice, and/or policy

The map points to several gaps in the evidence base with respect to primary studies. It also points to the lack of reviews and the methodological shortcomings in most existing reviews. Some of the implications for further research are:

- Efforts needed so that the key funders and researchers in the transport field reach the consensus to identify the priority area for research with weak evidence synthesis.
- Future research should study the interventions related to incentives and institutions and regulations in railways, port, shipping and waterways, and civil aviation.
- To fill the important gaps in this sector, there is a need for more studies on the areas of environment, education, culture, gender equity, and transport equity.
- The geographical base of evidence needs to be expanded, the majority of the studies to date are from East Asia and Pacific.

## CONTRIBUTIONS OF AUTHORS

The lead author is the person who develops and co‐ordinates the EGM team, discusses and assigns roles for individual members of the team liaises with the editorial base and takes responsibility for the ongoing updates of the EGM.

Content expertise:

Nina Blöndal has conducted several impact evaluations of transport interventions and authored a chapter on transport impact evaluation for the ADB Guidebook. Dr. Howard White co‐edited a special issue of the Journal of Development Effectiveness on infrastructure impact evaluations including contributing a paper on mixed methods in infrastructure studies.

SR method expertise:

All authors are experienced systematic reviewers, which means that they are proficient in conducting various processes in an EGM, such as screening, quality assessment and coding. Howard White will provide technical support for the conducting the review.

EGM methods expertise:

Howard White as CEO provides technical and strategic support for the development of the EGM.

All team members have previous experience in SR methodology, including search, data collection, statistical analysis, theory‐based synthesis, which mean they are proficient in carrying out the various processes in an EGM, such as search, eligibility screening, quality assessment and coding.

Information retrieval expertise:

John Eyers is a trained information retrieval specialist and has experience of supporting over 50 systematic maps and reviews in social sciences areas.

## DECLARATIONS OF INTEREST

Howard White is the CEO of the Campbell Collaboration. He has no role in the editorial process for this EGM.

## PLANS FOR UPDATING THE EGM

We plan to update the map (or support others in doing so) when sufficient further studies and resources become available.

## DIFFERENCES BETWEEN PROTOCOL AND MAP

None.

## SOURCES OF SUPPORT

This EGM is supported by the UK Foreign, Commonwealth and Development Office (FCDO) under its support for the Centre for Excellence for Development Impact and Learning (CEDIL).

## Supporting information

Supporting information.

## Footnotes

1
https://www.railjournal.com/africa/angola‐drc‐rail‐link‐restored‐after‐34‐years/.

2
https://knoema.com/atlas/ranks/Road‐density.

3 A brief overview of studies is given in https://blogs.lse.ac.uk/southasia/2013/04/29/railways‐and‐indian‐economic‐development/.

4
https://sustainabledevelopment.un.org/index.php?page=view&type=20000&nr=802&menu=2993

5
https://www.china‐briefing.com/news/china‐rail‐network‐10000‐km‐domestic‐expansion‐link‐key‐city‐clusters/.
