# Supplementary material for: Studies of the effectiveness of transport sector interventions in low‐ and middle‐income countries: An evidence and gap map
Source: Campbell Syst Rev. 2021 Nov 27;17(4):e1203. doi: 10.1002/cl2.1203 (PMC8724647; doi:10.1002/cl2.1203)
Supplement: Supplementary file 1 — Supporting information. [file CL2-17-e1203-s001.docx]

**Online supplements**

***List of online supplements***

Online interactive EGM

**Appendix A: Framework**

**Intervention categories and sub-categories**

| Category | Sub-categories | Examples |
| --- | --- | --- |
| Roads and pathways (including cycle paths) | Infrastructure | Construction and upgrading of roads, and highways  Infrastructure maintenance |
|  | Incentives | Road pricing and tolls  Subsidies and taxes |
|  | Institutions (including regulations) | Road legislation and agencies  Vehicle and driving regulations  Public private partnership (PPP) |
| Rail and trams | Infrastructure | Construction and upgrading  Maintenance |
|  | Incentives | Pricing structure  Subsidies to rail operators |
|  | Institutions (including regulations) | Regulatory framework  Public private partnership (PPP)  Nationalisation/privatisation |
| Ports, shipping and waterways | Infrastructure | Port and inland waterway construction and rehabilitation including modernization  Maintenance |
|  | Incentives | Tolls and other charges  Taxes and subsidies |
|  | Institutions (including regulations) | Port authorities |
| Civil Aviation | Infrastructure | Airports |
|  | Incentives | Taxes and subsidies |
|  | Institutions (including regulations) | Airport authorities |

*Outcomes*

| Domain | Sub-domain |
| --- | --- |
| Transport infrastructure, services and use | Infrastructure quantity  Infrastructure quality (inc. safety assessment)  Infrastructure services  Transport time or costs (inc. congestion and VOC)  Market access  Transport modality (inc. car ownership)  Safe practices |
| Economic Impact | Household income and poverty  Economic Development  Employment and migration  Trade and tourism  Location (land use) and prices  Displacement |
| Health and education | Access to health facilities  Health outcomes  Access to education facilities  Education outcomes |
| Culture | Values, language and social cohesion  Cultural heritage  Cultural diversity |
| Environment | Air quality  Noise pollution  Habitat destruction |
| Economic and equity analysis | Cost effectiveness or CBA  Gender equity  Transport equity^[[1]](#footnote-1)^ |

**Appendix B: Search terms**

**Intervention search term**

- DE "TRANSPORTATION" OR DE "AIR travel" OR DE "AUTOMOTIVE transportation" OR DE "BUS transportation" OR DE "CARRIAGES & carts" OR DE "CARRIERS" OR DE "CHOICE of transportation" OR DE "COMMUTING" OR DE "DEEP sea passenger transportation" OR DE "DELIVERY of goods" OR DE "DRAYAGE" OR DE "EMERGENCY transportation" OR DE "EMPLOYER-sponsored transportation" OR DE "FERRIES" OR DE "FERRY routes" OR DE "FREIGHT & freightage" OR DE "GROUND passenger transportation" OR DE "HARBORS" OR DE "HIGH speed ground transportation" OR DE "INTERNATIONAL transit" OR DE "OCEAN travel" OR DE "PASSES (Transportation)" OR DE "PUBLIC transit" OR DE "RAILROAD travel" OR DE "RAILROADS" OR DE "ROADS" OR DE "ROUTE surveying" OR DE "RURAL transportation" OR DE "SHIPPING (Water transportation)" OR DE "SHUTTLE services" OR DE "SUSTAINABLE transportation" OR DE "TAXI service" OR DE "TRANSPORTATION demand management" OR DE "TRANSPORTATION management system" OR DE "TRANSPORTATION of school children" OR DE "URBAN transportation" OR DE "VEHICLES" OR DE "WAGON trains" OR DE "WATERWAYS" OR DE "FINANCING of transportation" OR DE "PUBLIC transit commissions" OR DE "TRANSPORTATION accidents" OR DE "TRANSPORTATION agencies" OR DE "TRANSPORTATION departments" OR DE "TRANSPORTATION industry" OR DE "TRANSPORTATION laws" OR DE "TRANSPORTATION policy"
- (DE "inland transport" OR DE "international transport" OR DE "long distance transport" OR DE "air transport" OR DE "rail transport" OR DE "refrigerated transport" OR DE "road transport" OR DE "bus transport" OR DE "airports" OR DE "railways" OR DE "roads" OR DE "transport costs" OR DE "transporting quality" OR DE "water transport" OR DE "waterways" OR DE "transport") OR DE “rural transport”
- TI ( (Infrastructur* OR maintenance or maintain* OR repair* OR construction OR upgrade OR upgrading]) N6 (road* OR rail* OR tram* OR port OR ports OR ship* OR ships OR shipping OR waterway* OR aviation OR aircraft* OR "mass transport*" OR subway* OR transportation OR busway OR highway OR taxi* OR auto* OR "public trans*" OR "commuter trans*" OR "mass transit" OR "commuter train*" OR "passenger trans*" OR "passenger train*" OR trucks OR trucking OR freight OR lorry OR lorries OR vehicles)) ) OR AB ( (Infrastructur* OR maintenance or maintain*) N6 (road* OR rail* OR tram* OR port OR ports OR ship* OR ships OR shipping OR waterway* OR aviation OR aircraft* OR "mass transport*" OR subway*) ) OR SU ( (Infrastructur* OR maintenance or maintain*) N6 (road* OR rail* OR tram* OR port OR ports OR ship* OR ships OR shipping OR waterway* OR aviation OR aircraft* OR "mass transport*" OR subway*) )
- TI ( (Incentiv* OR price OR prices OR pricing OR tariff* OR toll*) N6 (road* OR rail* OR tram* OR port OR ports OR ship* OR ships OR shipping OR waterway* OR aviation OR aircraft* OR "mass transport*" OR subway*) ) OR AB ( (Incentiv* OR price OR prices OR pricing OR tariff* OR toll*) N6 (road* OR rail* OR tram* OR port OR ports OR ship* OR ships OR shipping OR waterway* OR aviation OR aircraft* OR "mass transport*" OR subway*) ) OR SU ( (Incentiv* OR price OR prices OR pricing OR tariff* OR toll*) N6 (road* OR rail* OR tram* OR port OR ports OR ship* OR ships OR shipping OR waterway* OR aviation OR aircraft* OR "mass transport*" OR subway*) )
- TI ( (institution* OR organiz* OR organis* OR regulat*) N6 (road* OR rail* OR tram* OR port OR ports OR ship* OR ships OR shipping OR waterway* OR aviation OR aircraft* OR "mass transport*" OR subway*) ) OR AB ( (institution* OR organiz* OR organis* OR regulat* * OR policy OR policies OR law OR laws OR legistlat* OR agencies OR "public private partnership" OR privatization OR privatisation OR nationalization OR nationalisation) N6 (road* OR rail* OR tram* OR port OR ports OR ship* OR ships OR shipping OR waterway* OR aviation OR aircraft* OR "mass transport*" OR subway*) ) OR SU ( (institution* OR organiz* OR organis* OR regulat*) N6 (road* OR rail* OR tram* OR port OR ports OR ship* OR ships OR shipping OR waterway* OR aviation OR aircraft* OR "mass transport*" OR subway*) )
- TI (public private partnership OR PPP) OR (transport) N6 (access* OR services) OR (HDM-4) OR (road OR bridge OR congestion OR emission OR planning) N6 (toll OR charge OR tax)

**Study design search terms**

- TI ( ((quantitativ* N5 synthes*) OR "mixed method*" or mixed‐method*) ) OR AB ( ((quantitativ* N5 synthes*) OR "mixed method*" or mixed‐method*) ) OR SU ( ((quantitativ* N5 synthes*) OR "mixed method*" or mixed‐method*) )
- TI ( (random$ or RCT or "double difference" or "regression discontinuity" or "propensity score" or matching or "comparison group" or "control group" or "instrumental variable*" or heckmann) ) OR AB ( (random$ or RCT or "double difference" or "regression discontinuity" or "propensity score" or matching or "comparison group" or "control group" or "instrumental variable*" or heckmann) ) OR SU ( (random$ or RCT or "double difference" or "regression discontinuity" or "propensity score" or matching or "comparison group" or "control group" or "instrumental variable*" or heckmann) )
- TI ( ("meta regression" or "meta synth*" or "meta‐synth*" or "meta analy*" or metaanaly* or meta‐analy* or metanaly* or "metaregression" or meta-regression or "methodologic* overview" or "pool* analys*" or "pool* data" or "quantitative* overview" or "research integration") ) OR AB ( ("meta regression" or "meta synth*" or "meta‐synth*" or "meta analy*" or metaanaly* or meta‐analy* or metanaly* or "metaregression" or meta-regression or "methodologic* overview" or "pool* analys*" or "pool* data" or "quantitative* overview" or "research integration") ) OR SU ( ("meta regression" or "meta synth*" or "meta‐synth*" or "meta analy*" or metaanaly* or meta‐analy* or metanaly* or "metaregression" or meta-regression or "methodologic* overview" or "pool* analys*" or "pool* data" or "quantitative* overview" or "research integration") )
- TI ( ((systematic* or synthes*) N3 (research or evaluation* or finding* or thematic* or report or descriptive or explanatory or narrative or meta* or review*)) or (map N3 (evidence or gap)) ) OR AB ( ((systematic* or synthes*) N3 (research or evaluation* or finding* or thematic* or report or descriptive or explanatory or narrative or meta* or review*)) or (map N3 (evidence or gap)) ) OR SU ( ((systematic* or synthes*) N3 (research or evaluation* or finding* or thematic* or report or descriptive or explanatory or narrative or meta* or review*)) or (map N3 (evidence or gap)) )

**LMIC search terms-**

- TI ( ("transitional countr*" or "emerging econom*" or "global south") ) OR AB ( ("transitional countr*" or "emerging econom*" or "global south") )
- TI ( (lmic or lmics or "third world" or "lami countr*") ) OR AB ( (lmic or lmics or "third world" or "lami countr*") )
- TI (low N1 middle N1 countr*) OR AB (low N1 middle N1 countr*)
- TI ( (low* N1 (gdp or gnp or "gross domestic" or "gross national")) ) OR AB ( (low* N1 (gdp or gnp or "gross domestic" or "gross national")) )
- TI ( ((developing or "less* developed" or "least developed" or "under developed" or underdeveloped or "middle income" or "low* income" or underserved or "under served" or deprived or poor* or "resource limited" or "resource constrained") N1 (economy or economies)) ) OR AB ( ((developing or "less* developed" or "least developed" or "under developed" or underdeveloped or "middle income" or "low* income" or underserved or "under served" or deprived or poor* or "resource limited" or "resource constrained") N1 (economy or economies)) )
- TI ( ((developing or "less* developed" or "least developed" or "under developed" or underdeveloped or "middle income" or "low* income" or underserved or "under served" or deprived or poor* or "resource limited" or "resource constrained") N1 (countr* or nation? or population? or world or state*)) ) OR AB ( ((developing or "less* developed" or "least developed" or "under developed" or underdeveloped or "middle income" or "low* income" or underserved or "under served" or deprived or poor* or "resource limited" or "resource constrained") N1 (countr* or nation? or population? or world or state*)) )
- TI ( (Afghanistan or Albania or Algeria or Angola or Argentina or Armenia or Armenian or Azerbaijan or Bangladesh or Benin or Byelarus or Byelorussian or Belarus or Belorussian or Belorussia or Belize or Bhutan or Bolivia or Bosnia or Herzegovina or Hercegovina or Botswana or Brazil or Bulgaria or "Burkina Faso" or "Burkina Fasso" or "Upper Volta" or Burundi or Urundi or Cambodia or "Khmer Republic" or Kampuchea or Cameroon or Cameroons or Cameron or Camerons or "Cape Verde" or "Central African Republic" or Chad or China or Colombia or Comoros or "Comoro Islands" or Comores or Mayotte or Congo or Zaire or "Costa Rica" or "Cote d'Ivoire" or "Ivory Coast" or Cuba or Djibouti or "French Somaliland" or Dominica or "Dominican Republic" or "East Timor" or "East Timur" or "Timor Leste" or Ecuador or Egypt or "United Arab Republic" or "El Salvador" or Eritrea or Ethiopia or Fiji or Gabon or "Gabonese Republic" or Gambia or Gaza or "Georgia Republic" or "Georgian Republic" or Ghana or Grenada or Guatemala or Guinea or Guiana or Guyana or Haiti or Honduras or India or Maldives or Indonesia or Iran or Iraq or Jamaica or Jordan or Kazakhstan or Kazakh or Kenya or Kiribati or Korea or Kosovo or Kyrgyzstan or Kirghizia or "Kyrgyz Republic" or Kirghiz or Kirgizstan or "Lao PDR" or Laos or Lebanon or Lesotho or Basutoland or Liberia or Libya or Macedonia or Madagascar or "Malagasy Republic" or Malaysia or Malaya or Malay or Sabah or Sarawak or Malawi or Mali or "Marshall Islands" or Mauritania or Mauritius or "Agalega Islands" or Mexico or Micronesia or "Middle East" or Moldova or Moldovia or Moldovian or Mongolia or Montenegro or Morocco or Ifni or Mozambique or Myanmar or Myanma or Burma or Namibia or Nepal or "Netherlands Antilles" or Nicaragua or Niger or Nigeria or Muscat or Pakistan or Palau or Palestine or Panama or Paraguay or Peru or Philippines or Philipines or Phillipines or Phillippines or "Papua New Guinea" or Romania or Rumania or Roumania or Rwanda or Ruanda or "Saint Lucia" or "St Lucia" or "Saint Vincent" or "St Vincent" or Grenadines or Samoa or "Samoan Islands" or "Navigator Island" or "Navigator Islands" or "Sao Tome" or Senegal or Serbia or Montenegro or Seychelles or "Sierra Leone" or "Sri Lanka" or "Solomon Islands" or Somalia or Sudan or Suriname or Surinam or Swaziland or Eswatini or "South Africa" or Syria or Tajikistan or Tadzhikistan or Tadjikistan or Tadzhik or Tanzania or Thailand or Togo or "Togolese Republic" or Tonga or Tunisia or Turkey or Turkmenistan or Turkmen or Uganda or Ukraine or Uzbekistan or Uzbek or Vanuatu or "New Hebrides" or Venezuela or Vietnam or "Viet Nam" or "West Bank" or Yemen or Zambia or Zimbabwe) ) OR AB ( (Afghanistan or Albania or Algeria or Angola or Argentina or Armenia or Armenian or Azerbaijan or Bangladesh or Benin or Byelarus or Byelorussian or Belarus or Belorussian or Belorussia or Belize or Bhutan or Bolivia or Bosnia or Herzegovina or Hercegovina or Botswana or Brazil or Bulgaria or "Burkina Faso" or "Burkina Fasso" or "Upper Volta" or Burundi or Urundi or Cambodia or "Khmer Republic" or Kampuchea or Cameroon or Cameroons or Cameron or Camerons or "Cape Verde" or "Central African Republic" or Chad or China or Colombia or Comoros or "Comoro Islands" or Comores or Mayotte or Congo or Zaire or "Costa Rica" or "Cote d'Ivoire" or "Ivory Coast" or Cuba or Djibouti or "French Somaliland" or Dominica or "Dominican Republic" or "East Timor" or "East Timur" or "Timor Leste" or Ecuador or Egypt or "United Arab Republic" or "El Salvador" or Eritrea or Ethiopia or Fiji or Gabon or "Gabonese Republic" or Gambia or Gaza or "Georgia Republic" or "Georgian Republic" or Ghana or Grenada or Guatemala or Guinea or Guiana or Guyana or Haiti or Honduras or India or Maldives or Indonesia or Iran or Iraq or Jamaica or Jordan or Kazakhstan or Kazakh or Kenya or Kiribati or Korea or Kosovo or Kyrgyzstan or Kirghizia or "Kyrgyz Republic" or Kirghiz or Kirgizstan or "Lao PDR" or Laos or Lebanon or Lesotho or Basutoland or Liberia or Libya or Macedonia or Madagascar or "Malagasy Republic" or Malaysia or Malaya or Malay or Sabah or Sarawak or Malawi or Mali or "Marshall Islands" or Mauritania or Mauritius or "Agalega Islands" or Mexico or Micronesia or "Middle East" or Moldova or Moldovia or Moldovian or Mongolia or Montenegro or Morocco or Ifni or Mozambique or Myanmar or Myanma or Burma or Namibia or Nepal or "Netherlands Antilles" or Nicaragua or Niger or Nigeria or Muscat or Pakistan or Palau or Palestine or Panama or Paraguay or Peru or Philippines or Philipines or Phillipines or Phillippines or "Papua New Guinea" or Romania or Rumania or Roumania or Rwanda or Ruanda or "Saint Lucia" or "St Lucia" or "Saint Vincent" or "St Vincent" or Grenadines or Samoa or "Samoan Islands" or "Navigator Island" or "Navigator Islands" or "Sao Tome" or Senegal or Serbia or Montenegro or Seychelles or "Sierra Leone" or "Sri Lanka" or "Solomon Islands" or Somalia or Sudan or Suriname or Surinam or Swaziland or Eswatini or "South Africa" or Syria or Tajikistan or Tadzhikistan or Tadjikistan or Tadzhik or Tanzania or Thailand or Togo or "Togolese Republic" or Tonga or Tunisia or Turkey or Turkmenistan or Turkmen or Uganda or Ukraine or Uzbekistan or Uzbek or Vanuatu or "New Hebrides" or Venezuela or Vietnam or "Viet Nam" or "West Bank" or Yemen or Zambia or Zimbabwe) ) OR ( (Afghanistan or Albania or Algeria or Angola or Argentina or Armenia or Armenian or Azerbaijan or Bangladesh or Benin or Byelarus or Byelorussian or Belarus or Belorussian or Belorussia or Belize or Bhutan or Bolivia or Bosnia or Herzegovina or Hercegovina or Botswana or Brazil or Bulgaria or "Burkina Faso" or "Burkina Fasso" or "Upper Volta" or Burundi or Urundi or Cambodia or "Khmer Republic" or Kampuchea or Cameroon or Cameroons or Cameron or Camerons or "Cape Verde" or "Central African Republic" or Chad or China or Colombia or Comoros or "Comoro Islands" or Comores or Mayotte or Congo or Zaire or "Costa Rica" or "Cote d'Ivoire" or "Ivory Coast" or Cuba or Djibouti or "French Somaliland" or Dominica or "Dominican Republic" or "East Timor" or "East Timur" or "Timor Leste" or Ecuador or Egypt or "United Arab Republic" or "El Salvador" or Eritrea or Ethiopia or Fiji or Gabon or "Gabonese Republic" or Gambia or Gaza or "Georgia Republic" or "Georgian Republic" or Ghana or Grenada or Guatemala or Guinea or Guiana or Guyana or Haiti or Honduras or India or Maldives or Indonesia or Iran or Iraq or Jamaica or Jordan or Kazakhstan or Kazakh or Kenya or Kiribati or Korea or Kosovo or Kyrgyzstan or Kirghizia or "Kyrgyz Republic" or Kirghiz or Kirgizstan or "Lao PDR" or Laos or Lebanon or Lesotho or Basutoland or Liberia or Libya or Macedonia or Madagascar or "Malagasy Republic" or Malaysia or Malaya or Malay or Sabah or Sarawak or Malawi or Mali or "Marshall Islands" or Mauritania or Mauritius or "Agalega Islands" or Mexico or Micronesia or "Middle East" or Moldova or Moldovia or Moldovian or Mongolia or Montenegro or Morocco or Ifni or Mozambique or Myanmar or Myanma or Burma or Namibia or Nepal or "Netherlands Antilles" or Nicaragua or Niger or Nigeria or Muscat or Pakistan or Palau or Palestine or Panama or Paraguay or Peru or Philippines or Philipines or Phillipines or Phillippines or "Papua New Guinea" or Romania or Rumania or Roumania or Rwanda or Ruanda or "Saint Lucia" or "St Lucia" or "Saint Vincent" or "St Vincent" or Grenadines or Samoa or "Samoan Islands" or "Navigator Island" or "Navigator Islands" or "Sao Tome" or Senegal or Serbia or Montenegro or Seychelles or "Sierra Leone" or "Sri Lanka" or "Solomon Islands" or Somalia or Sudan or Suriname or Surinam or Swaziland or Eswatini or "South Africa" or Syria or Tajikistan or Tadzhikistan or Tadjikistan or Tadzhik or Tanzania or Thailand or Togo or "Togolese Republic" or Tonga or Tunisia or Turkey or Turkmenistan or Turkmen or Uganda or Ukraine or Uzbekistan or Uzbek or Vanuatu or "New Hebrides" or Venezuela or Vietnam or "Viet Nam" or "West Bank" or Yemen or Zambia or Zimbabwe) )
- TI ( (Africa or Asia or Caribbean or "West Indies" or "South America" or "Latin America" or "Central America") ) OR AB ( (Africa or Asia or Caribbean or "West Indies" or "South America" or "Latin America" or "Central America") ) OR ( (Africa or Asia or Caribbean or "West Indies" or "South America" or "Latin America" or "Central America") )
- DE "Developing Countries" OR DE "Argentina" OR DE "Aruba" OR DE "Bahamas" OR DE "Bahrain" OR DE "Barbados" OR DE "Belize" OR DE "Bermuda" OR DE "Bolivia" OR DE "Bonaire" OR DE "Brazil" OR DE "British Virgin Islands" OR DE "Brunei Darussalam" OR DE "Cameroon" OR DE "Cayman Islands" OR DE "Chile" OR DE "China" OR DE "Christmas Island" OR DE "Cocos Islands" OR DE "Colombia" OR DE "Congo" OR DE "Cook Islands" OR DE "Costa Rica" OR DE "Cote d'Ivoire" OR DE "Crozet Islands" OR DE "Cuba" OR DE "Curacao" OR DE "Cyprus" OR DE "Dominica" OR DE "Dominican Republic" OR DE "Easter Island" OR DE "Ecuador" OR DE "Egypt" OR DE "El Salvador" OR DE "Falkland Islands" OR DE "Federated States of Micronesia" OR DE "Fiji" OR DE "French Guiana" OR DE "Gabon" OR DE "Gambier Islands" OR DE "Ghana" OR DE "Grenada" OR DE "Guadeloupe" OR DE "Guam" OR DE "Guatemala" OR DE "Guyana" OR DE "Honduras" OR DE "India" OR DE "Indonesia" OR DE "Iran" OR DE "Iraq" OR DE "Jamaica" OR DE "Jordan" OR DE "Kenya" OR DE "Kerguelen Archipelago" OR DE "Korea Democratic People's Republic" OR DE "Korea Republic" OR DE "Kuwait" OR DE "Least Developed Countries" OR DE "Lebanon" OR DE "Libya" OR DE "Malaysia" OR DE "Marquesas Islands" OR DE "Marshall Islands" OR DE "Martinique" OR DE "Mauritius" OR DE "Mayotte" OR DE "Mexico" OR DE "Midway Islands" OR DE "Mongolia" OR DE "Montserrat" OR DE "Morocco" OR DE "Namibia" OR DE "New Britain" OR DE "New Caledonia" OR DE "New Ireland" OR DE "Nicaragua" OR DE "Nigeria" OR DE "Niue" OR DE "Northern Mariana Islands" OR DE "Oman" OR DE "Pakistan" OR DE "Panama" OR DE "Papua New Guinea" OR DE "Paraguay" OR DE "Peru" OR DE "Philippines" OR DE "Algeria" OR DE "Puerto Rico" OR DE "Qatar" OR DE "Reunion" OR DE "Saba" OR DE "Saint Helena" OR DE "Saint Kitts and Nevis" OR DE "Saint Lucia" OR DE "Saint Vincent and the Grenadines" OR DE "Saudi Arabia" OR DE "Senegal" OR DE "Seychelles" OR DE "Singapore" OR DE "South Africa" OR DE "Sri Lanka" OR DE "Suriname" OR DE "Swaziland" OR DE "Syria" OR DE "Tahiti" OR DE "Thailand" OR DE "Tokelau" OR DE "Tonga" OR DE "Angola" OR DE "Anguilla Island" OR DE "Trinidad and Tobago" OR DE "Tuamotu" OR DE "Tubuai Islands" OR DE "Tunisia" OR DE "Turkey" OR DE "Turks and Caicos Islands" OR DE "United Arab Emirates" OR DE "Uruguay" OR DE "Venezuela" OR DE "Vietnam" OR DE "Wallis and Futuna" OR DE "Western Sahara" OR DE "Zimbabwe" OR DE "Antigua and Barbuda"
- DE "Caribbean" OR DE "Bahamas" OR DE "Turks and Caicos Islands" OR DE "Antilles" OR DE "French West Indies" OR DE "Guadeloupe" OR DE "Martinique"
- DE "Pacific Islands" OR DE "Macquarie Island" OR DE "Melanesia" OR DE "Micronesia" OR DE "Norfolk Island" OR DE "Polynesia" OR DE "Wake Island" OR DE "French Polynesia" OR DE "Gambier Islands" OR DE "Marquesas Islands" OR DE "Society Islands" OR DE "Tuamotu" OR DE "Tubuai Islands" OR DE "Oceania" OR DE "Australasia" OR DE "Micronesia" OR DE "Polynesia"
- DE "South East Asia" OR DE "Brunei Darussalam" OR DE "Indochina" OR DE "Indonesia" OR DE "Malaysia" OR DE "Myanmar" OR DE "Philippines" OR DE "Singapore" OR DE "Taiwan" OR DE "Thailand" OR DE "West Asia" OR DE "Armenia" OR DE "Azerbaijan" OR DE "Iran" OR DE "Iraq" OR DE "Israel" OR DE "Jordan" OR DE "Kazakhstan" OR DE "Kyrgyzstan" OR DE "Lebanon" OR DE "Afghanistan" OR DE "Oman" OR DE "Palestine" OR DE "Persian Gulf States" OR DE "Republic of Georgia" OR DE "Saudi Arabia" OR DE "Syria" OR DE "Tajikistan" OR DE "Turkey" OR DE "Turkmenistan" OR DE "Uzbekistan" OR DE "Yemen" OR DE "East Asia" OR DE "China" OR DE "Japan" OR DE "Korea Democratic People's Republic" OR DE "Korea Republic" OR DE "Mongolia" OR DE "South Asia" OR DE "Bangladesh" OR DE "Bhutan" OR DE "India" OR DE "Nepal" OR DE "Pakistan" OR DE "Sri Lanka" OR DE "Central Asia" OR DE "Kazakhstan" OR DE "Kyrgyzstan" OR DE "Mongolia" OR DE "Afghanistan" OR DE "Tajikistan" OR DE "Turkmenistan" OR DE "Uzbekistan" OR DE "Xinjiang"
- DE "Mexico"
- DE "South America" OR DE "Argentina" OR DE "Bolivia" OR DE "Brazil" OR DE "Chile" OR DE "Colombia" OR DE "Ecuador" OR DE "Falkland Islands" OR DE "French Guiana" OR DE "Guyana" OR DE "Paraguay" OR DE "Peru" OR DE "Amazonia" OR DE "Suriname" OR DE "Uruguay" OR DE "Venezuela" OR DE "Latin America" OR DE "Argentina" OR DE "Bolivia" OR DE "Brazil" OR DE "Chile" OR DE "Colombia" OR DE "Costa Rica" OR DE "Cuba" OR DE "Dominican Republic" OR DE "Ecuador" OR DE "El Salvador" OR DE "Guatemala" OR DE "Honduras" OR DE "Mexico" OR DE "Nicaragua" OR DE "Panama" OR DE "Paraguay" OR DE "Peru" OR DE "Puerto Rico" OR DE "Uruguay" OR DE "Venezuela" OR DE "Central America" OR DE "Belize" OR DE "Costa Rica" OR DE "El Salvador" OR DE "Guatemala" OR DE "Honduras" OR DE "Nicaragua" OR DE "Panama"
- DE "Africa" OR DE "Francophone Africa" OR DE "Africa South of Sahara" OR DE "North Africa" OR DE "Portuguese Speaking Africa" OR DE "Anglophone Africa"

## Appendix C: Coding Tool

| Study design | - - RCT   - Quasi-experimental study   - Cluster-quasi RCT   - Systematic Review   - Regression Discontinuity Design   - Controlled before and after study   - Cost-effectiveness analysis   - Cost benefit analysis   - Economic Impact   - Transport equity/justice |
| --- | --- |
| Publication status | - - Completed   - Ongoing |
| Study Methods | - - Difference in difference   - Propensity Score Matching   - Instrument Variable/Heckmann selection   - Multivariate/covariate adjusted analysis (e.g. ANCOVA analysis)   - Bivariate analysis/comparison of means |
| Population | - - Rural   - Urban   - Both rural and urban |
| Region | - - East Asia & Pacific   - Latin America & Caribbean   - Middle East & North Africa   - South Asia   - Sub-Saharan Africa   - Europe & Central Asia |
| Intervention | - Infrastructure   - - Roads     - Rail, trams, monorail     - Ports, shipping, and waterways     - Civil Aviation   - Incentives     - Roads     - Rail, trams, monorail     - Ports, shipping, and waterways     - Civil Aviation   - Institutions (including regulations)     - Roads     - Rail, trams, monorail     - Ports, shipping, and waterways |
| Outcome | - - Transport infrastructure, services and use     - Infrastructure quantity     - Infrastructure quality     - Infrastructure services     - Transport time and costs     - Market access     - Transport modality     - Safe practices   - Economic impact     - Household income and poverty     - Economic development     - Employment and migration     - Trade and Tourism     - Location, land use and prices     - Displacement   - Health and Education     - Access to health facilities     - Health outcomes     - Access to education facilities     - Education outcomes   - Culture     - Values, language and social cohesion     - Cultural heritage     - Cultural diversity   - Environment     - Air Quality     - Noise pollution     - Habitat destruction   - Economic & equity analysis     - Cost analysis inc CBA     - Gender equity     - Transport equity |
| Study type | - - Impact evaluations   - Systematic review |

**AMSTAR-2 for systematic reviews**

1. Did the research questions and inclusion criteria for the review include the components of PICO?
   - 1. Yes
        1. Population
        2. Intervention
        3. Comparator group
        4. Outcome
        5. Time frame for follow-up (optional)
     2. No
2. Did the report of the review contain an explicit statement that the review methods were established prior to the conduct of the review and did the report justify any significant deviations from the protocol?
   - 1. Yes: The authors state that they had a written protocol or guide that included ALL the following
        1. Review question
        2. Search strategy
        3. Inclusion/exclusion criteria
        4. a risk of bias assessment
        5. a meta-analysis/synthesis plan, if appropriate,
        6. a plan for investigating causes of heterogeneity
        7. justification for any deviations from the protocol
     2. Partial Yes: The authors state that they had a written protocol or guide that included ALL the following
        1. review question(s)
        2. a search strategy
        3. inclusion/exclusion criteria
        4. a risk of bias assessment
     3. No
3. Did the review authors explain their selection of the study designs for inclusion in the review?
   - 1. Yes: If the review satisfy ONE of the following
        1. Explanation for including only RCTs
        2. OR Explanation for including only NRSI
        3. OR Explanation for including both RCTs and NRSI
     2. No
4. Did the review authors use a comprehensive literature search strategy?
   - 1. Yes: Should have all the following
        1. searched at least two databases (relevant to research question)
        2. provided key word and/or search strategy
        3. justified publication restrictions (e.g. language)
        4. searched the reference lists / bibliographies of included studies
        5. searched trial/study registries
        6. included/consulted content experts in the field
        7. where relevant, searched for grey literature
        8. conducted search within 24 months of completion of the review
     2. Partial yes: All the following
        1. searched at least two databases (relevant to research question)
        2. provided key word and/or search strategy
        3. justified publication restrictions (e.g. language)
     3. No
5. Did the review authors perform study selection in duplicate?
   - 1. Yes, either ONE of the following
        1. at least two reviewers independently agreed on selection of eligible studies and achieved consensus on which studies to include
        2. two reviewers selected a sample of eligible studies and achieved good agreement (at least 80 percent), with the remainder selected by one reviewer.
     2. No
6. Did the review authors perform data extraction in duplicate?
   - 1. Yes: either ONE of the following
        1. at least two reviewers achieved consensus on which data to extract from included studies
        2. two reviewers extracted data from a sample of eligible studies and achieved good agreement (at least 80 percent), with the remainder extracted by one reviewer
     2. No
7. Did the review authors provide a list of excluded studies and justify the exclusions?
   - 1. Yes: if it includes the following
        1. provided a list of all potentially relevant studies that were read in full-text form but excluded from the review
        2. Justified the exclusion from the review of each potentially relevant study
     2. Partial Yes if:
        1. provided a list of all potentially relevant studies that were read in full-text form but excluded from the review
     3. No
8. Did the review authors describe the included studies in adequate detail?
   - 1. Yes: should also have ALL the following
        1. described population in detail
        2. described intervention in detail (including doses where relevant)
        3. described comparator in detail (including doses where relevant)
        4. described study’s setting
        5. timeframe for follow-up
     2. Partial Yes: should have the following
        1. described populations
        2. described interventions
        3. described comparators
        4. described outcomes
        5. described research designs
     3. No
9. Did the review authors use a satisfactory technique for assessing the risk of bias (RoB) in individual studies that were included in the review?
   - 1. RCTs
        1. Yes: must have assessed RoB from
           1. allocation sequence that was not truly random, and
           2. selection of the reported result from among multiple measurements or analyses of a specified outcome
        2. Partial Yes:must have assessed RoB from
           1. unconcealed allocation, and
           2. lack of blinding of patients and assessors when assessing outcomes (unnecessary for objective outcomes such as all-cause mortality)
        3. No
     2. NRSI
        1. Yes: must also have assessed RoB from
           1. methods used to ascertain exposures and outcomes, and
           2. selection of the reported result from among multiple measurements or analyses of a specified outcome
        2. Partial Yes: must have assessed RoB
           1. from confounding, and
           2. from selection bias
        3. No
10. Did the review authors report on the sources of funding for the studies included in the review
    - 1. Yes: Must have reported on the sources of funding for individual studies included in the review. Note: Reporting that the reviewers looked for this information but it was not reported by study authors also qualifies
      2. No
11. If meta-analysis was performed did the review authors use appropriate methods for statistical combination of results?
    - 1. RCTs
         1. Yes if
            1. The authors justified combining the data in a meta-analysis
            2. AND they used an appropriate weighted technique to combine study results and adjusted for heterogeneity if present.
            3. AND investigated the causes of any heterogeneity
         2. No
         3. No meta-analysis conducted
      2. For NRSI
         1. Yes if
            1. The authors justified combining the data in a meta-analysis
            2. AND they used an appropriate weighted technique to combine study results, adjusting for heterogeneity if present
            3. AND they statistically combined effect estimates from NRSI that were adjusted for confounding, rather than combining raw data, or justified combining raw data when adjusted effect estimates were not available
            4. AND they reported separate summary estimates for RCTs and NRSI separately when both were included in the review
         2. No
         3. No meta-analysis conducted
12. If meta-analysis was performed, did the review authors assess the potential impact of RoB in individual studies on the results of the meta-analysis or other evidence synthesis?
    - 1. Yes if
         1. included only low risk of bias RCTs
         2. OR, if the pooled estimate was based on RCTs and/or NRSI at variable RoB, the authors performed analyses to investigate possible impact of RoB on summary estimates of effect
      2. No
      3. No meta-analysis conducted
13. Did the review authors account for RoB in individual studies when interpreting/ discussing the results of the review?
    - 1. Yes if
         1. included only low risk of bias RCTs
         2. OR, if RCTs with moderate or high RoB, or NRSI were included the review provided a discussion of the likely impact of RoB on the results
      2. No
14. Did the review authors provide a satisfactory explanation for, and discussion of, any heterogeneity observed in the results of the review?
    - 1. Yes if
         1. There was no significant heterogeneity in the results
         2. OR if heterogeneity was present the authors performed an investigation of sources of any heterogeneity in the results and discussed the impact of this on the results of the review
      2. No
15. If they performed quantitative synthesis did the review authors carry out an adequate investigation of publication bias (small study bias) and discuss its likely impact on the results of the review?
    - 1. Yes if
         1. performed graphical or statistical tests for publication bias and discussed the likelihood and magnitude of impact of publication bias
      2. No
      3. No meta-analysis conducted
16. Did the review authors report any potential sources of conflict of interest, including any funding they received for conducting the review?
    - 1. Yes if
         1. The authors reported no competing interests OR
         2. The authors described their funding sources and how they managed potential conflicts of interest
      2. No
    1. Overall study quality
       1. High: No or one non-critical weakness *the systematic review provides an accurate and comprehensive summary of the results of the available studies that address the question of interest*
       2. Moderate: More than one non-critical weakness
       3. Low: One critical flaw* with or without non-critical weaknesses

**Appendix D Definitions of Interventions and Outcome**

| Terms | Definition |
| --- | --- |
| Transport infrastructure, services and use- Transport infrastructure (e.g. roads, railways, ports, or airports), upgrading existing links and technology, or improving transport services, such as public bus services. Services available, introduced and usage of the available transport infrastructure. | |
| Infrastructure quantity | Infrastructure increase or growth |
| Infrastructure quality (inc. safety assessment) | Quality of the available infrastructure (Road quality) |
| Infrastructure services | Logistics- transportation of the agricultural products, goods and other materials. |
| Transport time or costs (inc. congestion and VOC) | Access to transport infrastructure, travel time, time taken to access the available transport infrastructure, frequency of service, connectivity, travel cost and Congestion.  Congestion in transport is a major problem in both developed and developing countries involving high opportunity costs |
| Market access | Access to market by the population and it also include the access by the enterprises or farmers to sell their goods in the market. |
| Transport modality (inc. car ownership) | Modes of transportation and it include ownership (Car) |
| Safe practices | Safe practices such as speed limits, use of helmets and other practices. |
| Economic Impact- Economic impact analysis is an exercise to determine how a transport intervention project or policy affects the amount and type of economic activity in a region. Provision of transport as a service to reduce poverty by increasing economic efficiency and enhancing opportunities. Transport allows people to reach out to job or its effects on Employment opportunities and migration. | |
| Household income and poverty | Increase in household income, Poverty |
| Economic Development | Enterprise development (Profitability), GDP and Agricultural production |
| Employment and migration | Increase in Employment opportunities  Road accessibility has impact on population movements. |
| Trade and tourism | Transfer of goods and services, trade activities and tourism development and affects. |
| Location (land use) and prices | Locations of the firm or the household. Effects on the prices of the property. |
| Displacement | Displacement of the population due to transport infrastructure. (Construction, other infrastructure development projects) |
| Health and education- Transport can affect health, both positive (access to health services, higher income, availability of more diversified diet etc.) and negative (road traffic injuries, air pollution, and spreading disease).  Education also affected by the transport, it gives access to educational facilities and lack of transport infrastructures affect the educational status of the population. | |
| Access to health facilities | Health facilities- Health centres (Primary and secondary) and Emergency services (Obstetric) |
| Health outcomes | Health related outcomes- Improved health status, improvement in health conditions disease.  Spread of disease - Transport systems can also help spread infectious diseases, such as the recent Ebola epidemic (World Bank, 2014) and Covid-19  Road traffic injuries- Rise in fatalities and road injuries especially in LMICs due to poor quality of roads and road safety regulations |
| Access to education facilities | Education facilities such as Schools, college or vocational centres. |
| Education outcomes | Educational status – School enrolment, attendance rate, dropout rates. |
| Culture- Cultural effects, both positive and negative consequences of increased mobility within and between nations. | |
| Values, language and social cohesion | Effects on the social cohesion, values of the population (Due to forced displacement and migration) |
| Cultural heritage |  |
| Cultural diversity | Different cultural, its diversity |
| Environment- Transport system may also disturb ecosystem through deforestation, biodiversity loss, pollution, road kill, and blocking of seasonal migration patterns of wildlife | |
| Air quality | Air pollution caused by vehicle emissions, from increased traffic volumes. |
| Noise pollution | Sounds of Vehicle and Industrial areas (Transport Hub) |
| Habitat destruction | Habitat loss and habitat reduction due to improved transport infrastructure |
| Economic and equity analysis | |
| Cost Analysis inc CBA | CBA- Cost benefit/benefit-cost analysis is an exercise to determine the social welfare effects of transport sector interventions in comparison to costs. |
| Gender equity | Promoting women travellers, provision or benefits to women in transport infrastructure, Gender promotion. |
| Transport equity^[[2]](#footnote-2)^ | Transportation equity or justice usually refers to the fairness with which the impacts of transportation such as benefits and costs are distributed. Horizontal equity, also called fairness and egalitarianism, is concerned with the distribution of impacts between individuals and groups considered equal in ability and need; vertical equity is concerned with the distribution of impacts between individuals and groups that differ in abilities and needs, for example by income or social class (also called social justice, environmental justice and social inclusion) or in transportation ability and need otherwise known as universal design |

1. Transportation equity or justice usually refers to the fairness with which the impacts of transportation such as benefits and costs are distributed. Horizontal equity, also called fairness and egalitarianism, is concerned with the distribution of impacts between individuals and groups considered equal in ability and need; vertical equity is concerned with the distribution of impacts between individuals and groups that differ in abilities and needs, for example by income or social class (also called social justice, environmental justice and social inclusion) or in transportation ability and need otherwise known as universal design (Litman, 2018) [↑](#footnote-ref-1)
2. Transportation equity or justice usually refers to the fairness with which the impacts of transportation such as benefits and costs are distributed. Horizontal equity, also called fairness and egalitarianism, is concerned with the distribution of impacts between individuals and groups considered equal in ability and need; vertical equity is concerned with the distribution of impacts between individuals and groups that differ in abilities and needs, for example by income or social class (also called social justice, environmental justice and social inclusion) or in transportation ability and need otherwise known as universal design (Litman, 2018) [↑](#footnote-ref-2)
